# Perceptions of a Buruli ulcer controlled human infection model: *How, who,* and *why?*

**DOI:** 10.1371/journal.pntd.0012593

**Published:** 2025-02-05

**Authors:** Stephen Muhi, Simone Schmidt, Julia L. Marshall, Daniel P. O’Brien, Paul D. R. Johnson, James S. McCarthy, Euzebiusz Jamrozik, Joshua Osowicki, Timothy P. Stinear

**Affiliations:** 1 Department of Microbiology and Immunology, Peter Doherty Institute for Infection and Immunity, The University of Melbourne, Melbourne, Victoria, Australia; 2 Victorian Infectious Diseases Service, Royal Melbourne Hospital, Parkville, Victoria, Australia; 3 Walter and Eliza Hall Institute of Medical Research, Parkville, Victoria, Australia; 4 Department of General Medicine, The Royal Melbourne Hospital, Melbourne, Victoria, Australia; 5 Faculty of Engineering and Information Technology, The University of Melbourne, Melbourne, Victoria, Australia; 6 Department of Infectious Diseases, Peter Doherty Institute for Infection and Immunity, The University of Melbourne, Victoria, Australia; 7 Department of Infectious Diseases, Barwon Health, Geelong, Victoria, Australia; 8 Austin Health, Heidelberg, Victoria, Australia; 9 Ethox Centre, University of Oxford, Oxford, United Kingdom; 10 Tropical Diseases Research Group, Murdoch Children’s Research Institute, The Royal Children’s Hospital, Parkville, Victoria, Australia; 11 Infectious Diseases Unit, Department of General Medicine, Royal Children’s Hospital Melbourne, Victoria, Australia; 12 Department of Paediatrics, University of Melbourne, Victoria, Australia; 13 Victorian Infectious Disease Reference Laboratory (VIDRL), The Peter Doherty Institute for Infection and Immunity, Melbourne, Victoria, Australia; Yale University School of Medicine, UNITED STATES OF AMERICA

## Abstract

**Background:**

Infection with *Mycobacterium ulcerans* causes slowly progressive skin lesions known as Buruli ulcer (BU). An *M. ulcerans* controlled human infection model (MuCHIM) is likely to accelerate our understanding of this otherwise neglected disease, and may be an efficient platform for testing vaccines and other interventions. The aim of this study was to understand perceptions of this model across a range of key stakeholders in an endemic Australian community setting.

**Methods:**

We recruited young adults who live near an Australian BU endemic area but without a personal history of BU, clinicians involved in the management of BU, young adults with a personal history of a small, treated BU, and participants of any age with a demonstrated interest in public advocacy related to their personal BU lived experience. Participants reviewed an abridged version of the provisional protocol. A series of three focus groups were then conducted by video, and the transcribed text was analysed using reflexive thematic analysis to generate themes for exploration.

**Results:**

Participants universally valued the outcomes that MuCHIM might deliver. The predominant theme was that informed consent required fully transparent communication with potential participants regarding what their participation would involve, how it would impact their lives, and both the expected outcome and ‘worst-case scenario’. They also offered actionable recommendations on how best to communicate the tension between the expected outcome and the ‘worst-case scenario’ of disease associated with delayed diagnosis and comorbidity, as typically portrayed by the media. Participants recommended including images and testimonials from people who have had BU to support the conditions for informed consent. Focus groups also gave a clear sense of who they believed would volunteer for this type of research.

**Conclusions:**

This study offers valuable guidance regarding the content and presentation of information to inform potential participants, with focus group participants suggesting a multimodal approach of communication, including lived experience testimonials and clinical images of the expected outcome. This information will inform development of materials for enrolment to adequately communicate risks and expectations to potential study participants.

## Author summary

Buruli ulcer (BU) is a slow-growing skin infection caused by the bacterium *Mycobacterium ulcerans*. A new type of research study, called the *Mycobacterium ulcerans* Controlled Human Infection Model (MuCHIM), involves deliberately infecting consenting adult volunteers and treating them quickly. This approach could help us learn more about BU and test vaccines (or other preventative measures) and treatments. This study asked for the opinions of people connected to BU in Victoria, Australia. We invited adults living near BU-affected areas (but who had not had BU themselves), doctors who treat patients with BU, young adults who have had a small, treated BU, and advocates who have publicly shared their own personal BU experience. Participants reviewed a summary of the MuCHIM research plan and then joined focus groups on Zoom. We analysed and summarised their discussions. Participants saw the research as valuable but emphasised the need for clear, honest communication. They wanted potential volunteers to fully understand what the study involves, how it might affect their lives, and the possible risks, including the ‘worst-case’ scenario. Suggestions included using images and stories from real BU patients to help understand the study. Participants also shared their thoughts on who might volunteer for such research. This feedback will guide how we explain risks and expectations to future MuCHIM study participants.

## Introduction

Infection of the skin with *Mycobacterium ulcerans* causes slowly progressive lesions known as ‘Buruli ulcer’ (BU) [1]. Disease is geographically restricted to areas of Australia and Africa. The World Health Organization (WHO) has classified BU as a Neglected Tropical Disease, with the goal of accelerating improved strategies for prevention and treatment. A controlled human infection model of *Mycobacterium ulcerans* (‘MuCHIM’) in healthy adult volunteers [2,3], would advance our understanding of host-pathogen interactions in BU, and could be an efficient platform for evaluating vaccines [4], chemoprophylaxis, and novel therapeutics [5].

The aim of this qualitative study was to explore public perceptions of a proposed MuCHIM study, and thereby to inform MuCHIM design and implementation [6–8]. Public consultation is increasingly being advocated for in clinical trials [6], to inform the design of the trial protocol and the content of participant information material, ensuring a person-centred approach to the research. This step may be considered particularly important in ‘human challenge’ studies because they involve intentional exposure of healthy volunteers to the risks of infection [9]. Embedding public beliefs and values in the design of the MuCHIM project is key to building trust and confidence in the trial, which in turn facilitates enrolment, safe study completion and high quality data. The study design for a recently reported cutaneous leishmaniasis CHIM was significantly influenced by focus group research [10]. Such discussions stimulate the generation of unique viewpoints and potentially identify issues not previously anticipated by researchers, particularly from people with lived experience of the disease being investigated.

Public involvement is encouraged by international and Australian authorities. In 2020, the WHO released criteria for the ethical acceptability of a human challenge trial of SARS-CoV-2 [11]. One criterion included public consultation and engagement, including the incorporation of the views of challenge study participants or those who have expressed an interest in participating [11]. This criterion also recommends transparency, and reinforces the importance of responding to community concerns [11]. The Australian National Health and Medical Research Council (NHMRC) has also highlighted the value of consumer and community involvement [12] in research. The Australian Code for the Responsible Conduct of Research states that ‘appropriate consumer involvement in research should be encouraged and facilitated by research institutions and researchers’ [12]. These principles were therefore employed to facilitate the development and conduct of the MuCHIM project to strengthen partnerships with representative community stakeholders.

This study was designed to characterise the perspectives of stakeholders regarding the proposed model, and their reflections on a provisional study protocol, including perceived barriers to its implementation. We included key stakeholders in Victoria, Australia, including (i) young people with a small BU and lived experience comparable to the expected MuCHIM participant journey, (ii) young people living in or near an endemic area, who may be eligible to participate in a MuCHIM trial and who are also likely to benefit from the downstream impact of BU research, and (iii) clinicians who manage patients with BU. We also recruited (iv) local public ‘ambassadors’ whose advocacy related to their personal BU experience has raised awareness of the disease.

## Methodology

### Ethics statement

This study was approved by the University of Melbourne Human Research Ethics Committee (Reference Number: 2024-29460-53104-5), in accordance with the National Statement on ethical Conduct in Human Research (NHMRC) (2007) and the Note for Guidance on Good Clinical Practice (CPMP/ICH-135/95). Focus group 1 P#4 provided written informed consent to share their deidentified photographs with other focus group members and for publication.

### Participant selection and recruitment

Participant recruitment was a purposeful selection of stakeholders, including young adults (18 to 45 years of age) who live near (≤10 km) an Australian endemic area but without a personal history of BU (labelled ‘A’ in analysis), participants who are involved in the management of BU in Australia (‘B’), and participants with a personal history of a small (<5 cm), treated BU (‘C’). The young adult age criterion was designed to sample participants who could be eligible to volunteer for the MuCHIM study. Additional participants (without age restriction) were also purposefully invited to participate as ambassadors if they had a demonstrated interest in public advocacy related to their personal BU experience; this included those involved in promotional activities related to raising awareness, such as in the media (‘D’). In addition, participants also needed to express an interest in participating in a focus group, needed to be available to participate on the scheduled days and times of the focus groups, and needed an internet connection. The study aimed to recruit a total of 18 participants across a range of relevant backgrounds. In such research, sample sizes are not defined by statistical power; rather, the priority is the richness of the data generated by in-depth analysis. This study therefore aimed to recruit diverse stakeholder representatives across numerous focus group meetings to generate a richer and more meaningful dataset.

Participants were recruited using poster advertisements at the University of Melbourne, and circulation of recruitment-related information was unrestricted (including word-of-mouth). Additional participants were invited via email, telephone or in person using existing networks of the MuCHIM Study Steering Committee and members of the WHO Collaborating Center (WHO-CC) for *M. ulcerans;* no participant was affiliated with the MuCHIM project or the WHO-CC for *M. ulcerans* prior to their involvement in this study*.* Participants were offered a AUD$100 gift voucher to acknowledge the time required to contribute to this study, as determined using NHMRC guidance [13]. All participants provided written informed consent to participate. Participants were emailed an abridged draft of the provisional protocol, with at least two weeks to review the material (S1 Material). No other MuCHIM-related participant-facing information was reviewed by focus group participants.

### Data collection

A total of three focus group interviews were undertaken, with six participants per focus group. Each focus group contained three participants from category ‘A’ (n=9), and one participant each from categories ‘B’ (n=3), ‘C’ (n=3), and ‘D’ (n=3). Of the 18 participants, 9 were male and 9 were female; as sex differences do not affect the risk of BU, this aligns with both the community at risk, as well as the eligible MuCHIM participant population. Focus groups were conducted at monthly intervals between June – August 2024. This number of focus groups aligned with evidence suggesting that as few as 2–3 participants can generate the majority of themes [14]. Based on previous literature [10,15], we aimed to include multiple small focus groups, of six participants each, to support the generation of novel ideas, as some participants may feel less comfortable voicing their opinions in larger group settings. We designed each focus group to comprise a mixture of relevant stakeholders, rather than grouping participants together based on eligibility criteria.

Data were collected using the in-depth semi-structured focus groups via videoconference software (Zoom, Version: 6.0.11). Data collected during the focus groups included the verbal discussions and any observational (non-verbal) communication (e.g., tone and emotion) that was observed by the facilitators, in order to provide an additional dimension of data for interpretation. A semi-structured discussion guide (S2 Material) with several open-ended questions was prepared based on a literature review regarding public perceptions of CHIM studies, particularly those involving the skin. The guide was then further developed following consultation with the MuCHIM Study Steering Committee (all investigators of this study). Participants with a previous history of BU were invited to use their lived experience to further generate questions or topics for discussion, and participants were encouraged to interact and engage with one another during the meeting.

The interviews were recorded via the videoconference platform hosted by the University of Melbourne. The focus groups were held during the week in the afternoon. Participants were invited to leave their videos turned on to maximise engagement. Participants provided written consent to being voice-recorded, and a second (back up) recording device (a smartphone voice recorder) was also used. Participant study data (audio file, transcripts, and analysis) were de-identified by assigning a unique study identifier for each participant. Data were stored on the University of Melbourne secure cloud server, with two factor authentication password protection making it only accessible to relevant study investigators. All recorded data were stored in mp3 (audio) format for text transcription and analysis. Back-up recordings were deleted after confirmation that the primary recording was successful and complete. Video recordings were converted into audio-only files for storage.

There were two focus group facilitators; as informed by a prior CHIM focus group [10], given the complex nature of the research and the importance of providing clarity, a principal investigator on the MuCHIM project (SM) acted as a co-facilitator. In addition, an independent external researcher (SS), with experience in qualitative research, acted as a co-facilitator to ensure impartial questioning to participant query. SM also maintained a reflexive journal to record and recognise internal motivations during the recruitment of participants, and during each focus group. The independent researcher double coded the transcribed data and performed thematic analysis to provide a novel lens and additional richness through the collaboration.

A form of data that was also collected included an overall statement of agreement (or disagreement) between members of each focus group, using a closed-ended question directed at the end of the discussion. Any disagreement was then recorded. These questions also aimed to clarify if the facilitators had appropriately captured the focus group’s perspectives, and to ensure no misinterpretation had occurred. Finally, all silences or lack of discussion were also documented as a form of data, potentially indicating overall consensus, lack of familiarity with the concept, or lack of engagement with the subject itself, which are all valuable forms of data for interpretation [15].

### Data analysis

Reflexive thematic analysis (RTA) with an experiential orientation was employed to analyse the data [16]. This analytic method, developed by Braun and Clarke, holds that knowledge is generated through researcher and participant collaboration [17,18]. Focused on participant experience and perspectives, this study is epistemologically positioned as contextualist. SM’s background in medicine, and SS’s background in the humanities, informed the analysis.

The in-built Zoom transcription software was utilised to create the initial text; SM corrected errors by reviewing the data manually alongside a recording of the focus group (this had the added benefit of familiarising SM with the data). SM and SS coded the data in NVivo, employing semantic coding, describing participant perspectives. Themes were generated from the codes rather than through an *a priori* conceptual framework. The researchers discussed their respective codes, locating points of resonance. After completion of the second focus group, SM and SS began to generate themes in collaboration from their existing codes and refined the themes through an iterative collaborative process.

### Analysis

**Fig 1:**
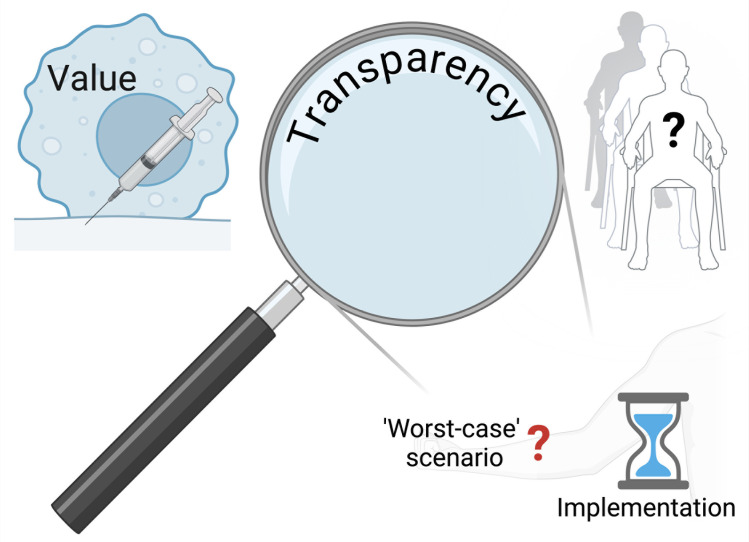
Themes included the importance of transparency (theme 1), including an exploration of what would constitute informed consent, and the concept of the ‘worst-case scenario’ (subtheme 1), and an exploration of the broader issues related to the protocol’s implementation, again framed by the importance of transparency in how the study may impact participants in their lives (subtheme 2). Other themes included *who* would participate in this model (theme 2), and finally, perceptions of the overall *value* of the research (theme 3). The relatively larger icon for ‘transparency’ represents its central importance as a critical theme. Created with BioRender.com.

The most critical and conceptually rich theme generated was the concept of ‘**transparency’** in communication with potential participants, regarding what their participation would involve, what impact it would have on their lives, and what they should expect the outcome of infection to be. Other themes generated, albeit with less emphasis, included understanding ‘**who’** would participate in a BU challenge model, and overall reflections on the ‘**value’** of the model. These themes are illustrated in Fig 1; the relatively larger icon for ‘transparency’ represents its central importance as a critical theme.

#### Theme 1: Transparency in communication with potential participants.

The overwhelmingly prevalent concern raised by participants was that ethical recruitment for this model would require potential participants to be fully informed of ‘what to expect’ and the ‘worst-case scenario’ of the ulcer and its subsequent scarring (as explored in Theme 1.1) as well as the implementation of the model and its impact on their life (as explored in Theme 1.2). Participants considered how best to communicate these aspects of *what to expect* and *the worst-case scenario* of infection and scarring as a clear pathway to obtaining informed consent.

#### Expected versus ‘worst-case’ scenarios.

Participants acknowledged that the media typically reported what may be considered the ‘worst-case scenario’, and this could frame public perceptions of the disease:

Focus group (FG) 1 P#1 (A): It seems like it’s very sensationalised […] So is a lot of the wording used to describe the disease.

Most participants considered that, although potential participants should be aware of the worst-case scenario outcomes of infection and treatment, participant information material should focus on the expected outcome of participation. For example,

FG2 P#6 (D): I think you should […] touch on [the worst-case scenario] and give some sort of indication of the likelihood of that, or situations in which those types of things can occur. A mention of it, but I wouldn’t focus on what you wouldn’t expect.

FG2 P#2 (A): I would lean towards describing […] what you’d expect […] the majority of people will go through if they participate in the study. There’s going to be many, many different ways things can go wrong […] like severe responses to antibiotics that are not common. And I don’t think you need to describe them […] but just noting that they can happen, just to have some more detail from [people with] lived experience and what you’re most likely to go through, would be useful.

Participants FG2 P#1 (A) and FG3 P#1 (A) considered how the ‘worst-case scenario’ of having the infection could be referred to in the material as a way of justifying the value of the research or ‘why’ this model is being implemented, but the information material should focus on what to expect from participation. FG3 P#1 suggested that the expected outcome of participation could be communicated to the potential participant as the purposefully designed result of the controlled parameters of the model. It is significant that this participant reported working in marketing/communication, for they reflected how to most meaningfully communicate this balance of information to the potential participant.

FG3 P#1 (A): If it was described to someone who would volunteer for this, they would Google what it really looks like, and you will get the worst-case scenario. You will see something that looks visually quite confronting. So, I think there’s no point denying [it]. But really outlining, based on the parameters of this trial, like in the protocol. There’s a lot of monitoring […] and you start the treatment early. With Buruli, […] ‘this’ is something that *can* happen. But with the way we’re planning on doing it, we don’t want it to get more than ‘that’ big. I think that’s a really clear point in recruiting people is acknowledging what can happen, and *that’s why this trial is really important*. Saying, ‘here’s what we’re trying to control,’ but that it won’t get too big in the course of this trial.

Another participant, who is a clinician who treats BU, FG3 P#4, agreed that this was the appropriate way of providing informed consent for potential participants in this model. They explained that there is negligible risk in this model in terms of developing the worst-case scenario of the ulcer.

FG3 P#4 (B): As someone who knows a lot about the disease, I would think that there’s a very small risk of […] a big inflammatory lesion causing a lot of pain and discharge and slow healing. I’d be more concerned that the model would actually just fail in a lot of people. You wouldn’t actually get an infection at all. […] If it’s done under such controlled circumstances, I think that the main reason we see such bad disease, like the others in this group are talking about with their personal experience, is because of the delay to diagnosis. But *if you started someone on antibiotics pre-emptively with a small nodule, […] it’s fairly low risk*.

Another clinician who treats BU, FG2 P#4 stated

I guess you do have to describe [… the] spectrum so long as […] the risks are properly described *because they’re under such close observation in this study you would think it’s unlikely you’re going to get a very large lesion that would require extensive surgery, or really extensive antibiotic therapy* […], so long as it’s properly framed.

For focus group participants, *proper framing* meant emphasising the expected outcome of the small ulcer under controlled parameters, and referring to the worst-case scenario of large lesions to justify the model, in order for potential participants to provide informed consent.

Participants considered how the expected outcomes of participation in the model could be communicated to potential participants via photographs and lived experience testimonials, which could help alleviate concerns and provide transparency regarding the impact of participation. For example,

FG2 P#2 (A): [P#6 and P#5…], they’ve obviously got experiences that I don’t have, that could be really useful for people that are considering participating. And so, could there […] be a paragraph […on] people’s lived experiences of […] these diseases and […] the treatments […] because it could really guide people’s decision making [… and] to have them shown […] pictures [of] what to expect before agreeing to participate.

FG3 P#6 (D), who had what could be referred to as the ‘worst-case scenario’ ulcer, due to delay in diagnosis and their pre-existing condition of diabetes, stated,

I think that if I saw photos of what my foot looked like, and I thought that that was going to happen to me, I wouldn’t be interested. If you showed me how it started off small, and that’s as far as it’s going to get, I’d do it.

This perspective demonstrates the importance of focusing on the expected outcomes of participation in the participant information material rather than focusing on the worst-case scenarios that are likely the outcome of delayed diagnosis and treatment, and comorbid risk factors, all of which are controlled by the nature of the MuCHIM trial.

In response to participant concerns regarding the ulcer and its subsequent scarring, a participant from FG1, P#5 (C) shared images of their BU journey:

This image was then shared with FG2 and FG3 participants, who considered it a useful way to demonstrate what to expect from participation in the model. For example, FG3 P#5 (C) stated,

Something like that photo you showed before with the three different stages […] would have been helpful for me because I Googled and saw the really horror story things and then I didn’t really see what a resolved ulcer looked like. So, perhaps something like that.

Some participants were concerned about scarring, especially from FG1, but overall, most participants expressed a lack of concern regarding scarring when informed that the scar would be small and minimally visible. For example:

FG1 P#3 (A): Reading the protocol, I thought, ‘Oh, but I could have a scar on my lovely skin’, I don’t think that I would do this research, which is so bad of me, because I know how important research is.

FG1 P#3 (A): Hearing from P#5 about how small it can actually be - and I’ve also got pale skin - and that it becomes not as visible, and it’s not really something that other people are going to notice […], as P#5 said, it’s so small you can’t even see [it] anymore. So, I think that’s eased my thoughts on that.

FG2 P#5 (C): Having a scar now is really not a big deal. I couldn’t care less, and barely look at it or think about it.

FG3 P#1 (A): I do have darker skin than most people and I personally wouldn’t really care. [...] I’ve got all sorts of random scars all over the place, because I injure myself all the time […] It’s not something that would particularly impact my decision. I think, as P#2 mentioned, it’s a cool story, if you know somebody’s like, ‘Oh, what’s that scar?’

#### Implementation of the model and its impact on participants.

Participants considered that all aspects of the protocol’s implementation would need to be transparently communicated to potential participants. Participants considered its importance regarding the impact of antibiotics, the rationale for the selected challenge site, the choice between surgical excision versus non-surgical approaches, and the expectations for potential participants’ involvement throughout the trial, particularly the lengthy time commitment required.

A key concern for participants was the effect of the antibiotics, and how this may influence their decision to undergo surgical excision of the lesion. Participants who had previous BU described the highly negative impact of being on antibiotics. For example,

FG2 P#6 (D): The side effects of those antibiotics are horrible. I had terrible rashes all over my body.

FG3 P#5 (C): I took my course of antibiotics in September last year, and I still don’t feel fully recovered.

Participants emphasised that it was important to convey to potential participants the impact of these antibiotics, for example, by these lived experience testimonials. Participants considered that transparency regarding the impact of the antibiotics could help potential participants in the model make an informed decision regarding whether to excise the ulcer, which would reduce the duration of antibiotics needed. Participants universally reported that they would opt for surgery, if they could reduce the amount of time on these antibiotics:

FG1 P#5 (C): Yeah, I think less antibiotics the better in this scenario, because […] they made me feel so crap.

FG1 P#1 (A): Maybe I would have more peace of mind if it was just excised and then just on antibiotics […] From what [… participants with BU] have said, the fact that these antibiotics can be pretty strong stuff, or they can have some side effects, and [cause] a lot of lifestyle changes […]. It would probably be something that I would prefer, to have surgery […] and then being on antibiotics for a less amount of time.

FG2 P#6 (D): I would go the surgical option with reduced antibiotic time, just because the antibiotics, in my experience with them, was not very nice.

Despite surgery being the unanimously favoured approach, participants still suggested that it would be important to provide the potential participant an alternative option, if available:

FG3 P#2 (A): It is good to have the choice. […] it’s always good for people to feel that it’s not a course that they don’t have much of a say in. […] The only way out is through, so if you have more options on what getting through that looks like to you, […] people will feel more confident about being involved. And I think, knowing that it’s comparable, say, to the excision of a suspicious mole, […] people would potentially feel that that’s a quicker fix, and […] possibly that would be a preferred option to a lot of people. But not everyone.

Most considered that inside the forearm was a good site because it can be easily monitored by the potential participant.

FG1 P#3 (A): I think that site would be fine. […] it’s the least impactful […] I think if it was higher, that would rub more, and then there [points to site] feels good […] It’s a good spot for us to be able to look at it and notice if something’s wrong, especially given that it isn’t painful, like if it was on the leg or where P#5 had [BU], I can imagine it’s not somewhere you look at all the time. If there were any changes that you needed to let researchers know about, it’s probably one of the better ones to be looking at. Also, I believe you said you’re going to want the participants take pictures, and it’s just an easy place for you to be able to take a picture yourself.

There was some contradiction in perspectives regarding the proposed site’s visibility to others. For example, one participant considered this a good location because it is not easily visible to others. Whereas other participants thought perhaps this site was less ideal because it is more easily visible to others than if the proposed site was the leg, and this visibility may cause anxiety in the participant.

FG3 P#2 (A): I think the location that you’ve chosen [...] I’m just looking at my posture now, and I do think that’s somewhere that’s not particularly evident.

FG2 P#5: It it’s visible in any way, then that’s probably not going to be ideal if you’re somebody who suffers from […] anxiety.

FG2 P#3: I also thought that was quite visible. And if people do have bandages for […] a couple of weeks they might get a bit put off by people constantly asking, ‘What’s wrong? What’s going on with your arm like that?’ […] That could be what triggers people to having negative feelings about it. […] So maybe somewhere less visible might be better, but still visible enough for the monitoring purposes.

Some participants, particularly clinicians, queried *why* the lower limb was not chosen for the challenge site, given this is the location where infection more commonly occurs. For example,

FG3 P#4 (B): The most common site tends to be the lower limb […]. Would you have a greater chance of success having a lower limb model? And would people care less about the scarring if it was more discreet on their legs rather than on their arm? I have very supple, beautiful skin on my forearms, but my legs are like P#1’s, covered in scars from my lack of coordination, so [...] I just wonder, was there any particular reason you went with the upper limb rather than the lower limb?

Researcher SM explained that the site was chosen because, as advised by specialists in the field, the skin of the forearm will heal more effectively after lesion excision. This explanation generated positive perceptions of the chosen site and reveals how deeply the participants value transparency in this research. For example, FG2 P#4, a clinician who treats BU and had queried the chosen site stated, ‘If the surgeons and dermatologists feel that the forearm gives the best result, then that does sound quite convincing to choose that site.’

Another key concern was the time commitment for potential participants in the model, and the impact on their lifestyle. As FG1 P#5 (C) stated: ‘I think possibly one of the hardest things is with the age demographic of 18 to 45 is the time commitment.’ Participants considered how this time commitment would need to be clearly communicated in participant information material. Participants who previously had BU described how treatment was time intensive and impacted their lives greatly. For example,

FG1 P#5 (D): It really was, in my words, a ‘time robber’, as far as treatment goes […].

FG1 P#6 (C) I had to take lots of chunks of time off work or out of Uni […], especially if you’re part of a trial for an extended period of time, and you’re having to come in for all these different checks and monitoring, it’s quite a significant time commitment.

Other participant concerns regarding potential participation included abstaining from alcohol, restricted travel, the duration of contraception, and the duration that they would need to monitor themselves for infection. Participants considered how all these aspects would need to be clearly communicated in the participant information material in terms of how the potential participant would be impacted. Ultimately, all participants agreed that the participant information material would need to be as transparent as possible regarding the impact on the potential participant’s life. To echo FG3 P#5 (C), the participant information material would need to convey ‘the impact of participating […], how it affects my work life, my home life [etc]’.

Finally, participants suggested that, although person-to-person transmission was not known to occur with current research evidence, this was still a concern of some participants, again framed by the context of maximising transparency and informed consent:

FG1 P#1 (A): You said in the protocol, there’s no human-to-human transmission. But what other kind of considerations might I have to take in my everyday life to make sure that I’m keeping [not only] myself safe, but also people around me safe?

FG2 P#2 (A): Maybe [be] a bit more explicit just to make it clear that if any family member were to develop a lesion, you would want to know.

#### Who would participate in this research?.

Participants considered the types of people who would participate in this model. Suggestions included altruistic individuals, people personally interested in research, and people who knew people who had been infected with a small lesion. On the other hand, although an individual may know someone who had had BU, they may not choose to participate if the shared experience was of the ‘worst-case scenario’. For example,

FG1 P#5 (C): People like my family, or people that have seen someone with it, and probably know that it cannot be too bad, and it also could lead to a really great outcome which is disease prevention through vaccines.

FG1 P#3 (A): I think that might be cancelled out a little bit if you know somebody that’s had a really bad one. And it’s been awful because I personally know someone where it didn’t get diagnosed for ages. And then it went to the bone, it’s been terrible.

Additional suggestions included people who live close to BU hotspots, adventurous individuals who want to tell a story, and those with the time to participate. It was also recognised that contraception requirements may dissuade people of childbearing potential:

FG2 P#6 (D): I also thought you probably won’t get as many females coming forward to participate. And then that could mean a male-dominated study. Would that be an issue?

FG1 P#6: (D) It’d be the adventurous types, that may be willing to sort of wear, dare I say, a badge on their arm that they’ve been down this road.

FG1 P#1 (A): There’s a cool story to tell in the sense of being part of a ‘flesh eating bacteria’ study. I think you’re going to find people who just generally want to have it as a story to tell.

A common idea generated in each focus group was that participants would be likely to have an interest in biomedical research. It was also suggested that those who had received a biomedical education would potentially also be more enabled to provide informed consent. These participants may also be likely to favour a surgical approach to treatment, in order to offer the greatest biological tissue for analysis. For example,

FG2 P#3 (A): You would also have a group of people who are quite literate in science and in research, and can see the benefit of a human challenge model.

FG1 P#5 (C): I think, if you are signing up for the trial, then you obviously already have some kind of interest in finding some kind of cure or vaccine, or some change to the current state of play. So, I think that would only probably make them more interested in also just having it biopsied or excised, […] knowing that you’re already in it.

Financial incentives were recognised as important, particularly in the context of the time dedication required to participate, and any potential interference with employment.

FG3 P#6 (D): Money makes a big difference […] I’ve just been involved with a lot of trials, and I like doing them. If I didn’t think I was going to get very sick [and…] this would just be a small thing, and if it possibly got worse, have it cut off […] I do think I’d do it. I’m just that sort of person, I suppose.

FG3 P#5 (C): Not only do they have to be altruistic and willing to do it […] it’s got to be people who can put in the time even just to show up […] People have jobs and kids and work and life and all that sort of stuff going on [...] that’s why I wondered about what kind of group that you would put it out to.

FG1 P5 (C): If you do react like I did to the antibiotics, does that mean you have to take days out of your sick leave, even though […] you’re sick because you’re part of a trial?

Although it was considered that it would be difficult to find potential participants for this study, as many would be ‘put off’ by being infected with an ulcer, and perhaps scared by alarmist media narratives, participants also noted that, given the small number of participants required for the model, it would be possible to generate the target sample.

#### Perceived value of the model.

Overwhelmingly, participants perceived the model to be of value. They explained that, as the ulcer is a problem of relevance to them, and there is need to develop better interventions for it, and because the infection is well suited to this model, given that effective treatment for it exists, this model is both feasible and important. For example,

FG1 P#1 (A): It sounds like interesting research […] and […] really important for vaccine development, and stuff like that.

FG1 P#5 (C): I think it’s really important. I think the work that you’re doing matters.

FG2 P#6 (D): I don’t think I’d want to have it again, but the fact that it’s being monitored and it’s treatable. We have to do these things to be able to develop treatments and improve them. And then […] the broader benefits of what this type of research could bring.

FG3 P#4 (B): It’s not a life-threatening illness. […] But it’s obviously something that causes great morbidity. So, it’s probably a prime candidate for something like this. And we have highly successful treatment for it […]. So, I think that this is the sort of disease that you would definitely target a human challenge model with.

FG3 P#3 (A): I work in a marketing communication sort of role. So, I’m thinking about how this would be represented in the public, the media and things like that, and I think right now with the fact that there has been quite a bit of media around Buruli […] the public perceive that this is a problem […]. So, a trial to try and address that problem is a good thing.

However, some participants enquired about alternatives to this model, whether the model was necessary given that effective treatment exists and also considered that there are other infections with higher incidence that do not have a CHIM or vaccine, and so questioned the model’s role in this respect.

FG1 P#3 (A): Is there a feasible alternative? Like better education about early identification of Buruli?

FG2 P#6 (D): It seems to be treatable, and the cases within Victoria, at least, I think the maximum is a few hundred cases reported. Potential perceptions, could be, ‘is it worth being vaccinated against this type of bacteria? Is it worth all the development?’ There are higher occurrences of this in perhaps developing countries and things like that, so that’s probably where the benefit is.

FG2 P#3 (A): I can see people probably having the perception that there are many other bacterial and viral infections that have a higher incidence, and yet don’t have vaccines under development and don’t have human challenge models. So, they might be wondering why a relatively neglected pathogen has this interest.

## Discussion

The exploration of stakeholder perceptions of MuCHIM informed investigators about the need to prioritise communication to enable fully informed consent to participation and appropriately target participant recruitment. An important perspective raised by the focus groups was how to effectively communicate the tension between describing the ‘worst-case scenario’, as represented by the media, and ‘what to expect’ from the minor infection proposed in the model, to provide potential participants with the appropriate information for their informed consent. Transparency, in terms of full disclosure of relevant information to participants, is an established ethical condition for fully informed consent, and the need for clarity has been identified in previous CHIM research [10,19]. The results of this study affirmed the importance of current processes and clarified more precisely aspects of the trial where participants sought greater clarity. These results will meaningfully inform the development of participant information sheets, by incorporating a clearer rationale for the selection of the upper limb as the challenge site, greater assurance regarding the lack of evidence of person-to-person transmission, and individualised and detailed information about how their involvement may impact their lives. But most importantly, the results of this study enable investigators to understand how to balance the complex tension of information regarding what is expected to happen to the participant in the model’s controlled environment, and what could happen to a human when infected with the pathogen in the general community.

Participants suggested using a multi-modal approach to achieving fully informed consent. Descriptive photographs and patient testimonials of the expected patient journey will therefore be collated for inclusion in patient information material. There is a paucity of literature describing the role of patient testimonials in obtaining informed consent [20]. Although more intensively researched, the role of visual aids in obtaining informed consent remains largely unclear, although researchers suggest that triallists should continue to explore innovative methods of providing information to potential participants [21].

**Fig 2:**
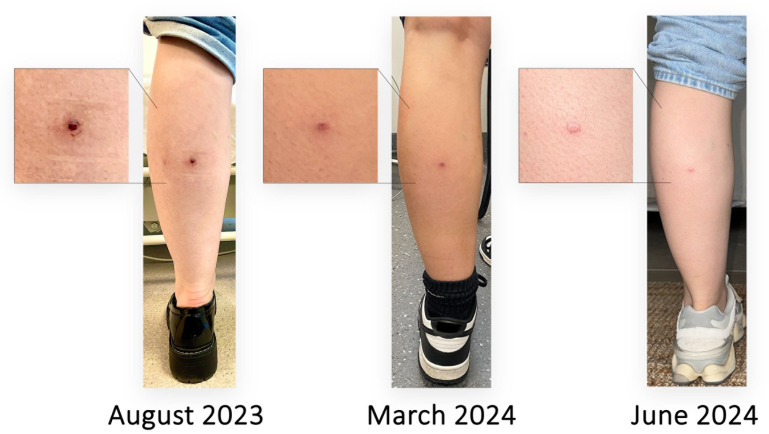
Focus group participant with a small (~3 mm) Buruli ulcer volunteered these images of their experience to demonstrate the clinical progress and scar formation of a small lesion diagnosed by punch biopsy, and was cured with 2 weeks of rifampicin/clarithromycin. The images were offered to demonstrate to candidate MuCHIM participants the expected progression and scar formation over time.

We hoped that including a diverse range of perspectives in each focus group would enable participants to generate a rich network of attitudes and ideas. Indeed, the interactions between representative participants produced an additional dimension for analysis. For example, participants who were initially concerned about the impact of scarring were reassured when a participant with a very small (promptly diagnosed) lesion reported their lived experience of healing. Similarly, when stories of antibiotic-related side effects were shared, this generated consensus that surgical excision would be the preferred approach for participants. This was further strengthened by participant advice to include such testimonials in potential participant related information, including the representative clinical images in Fig 2. By iteratively adapting to the ideas and suggestions generated in FG1 during FG2 and FG3, we validated the impact of utilising these clinical images as part of the information and consenting process to demonstrate the most likely outcome to potential participants.

Participants reported that all aspects of the protocol’s implementation would need to be transparently communicated to potential participants, including the rationale for the selected challenge site, the lengthy timeframes required, the impact of antibiotics and the choice between surgical and non-surgical treatment approaches. Aligned with the results of the focus group exploring the cutaneous leishmaniasis CHIM [10], we also found that participants favoured surgical excision. However, this was largely driven by negative participant experiences with antibiotics, and the desire to abbreviate the course of antibiotics needed. We hypothesise that including these testimonials in participant information material will influence participants towards excisional surgical approaches that minimise the need for prolonged courses of antibiotics. Nevertheless, participants suggested that autonomy for MuCHIM participants is important after being infected, when ‘the only way out is through’. Therefore, in the trial protocol, we intend to continue to provide participants with a non-surgical option, while informing them of their options using the lived-experience testimonials obtained in this study. The descriptions of antibiotic intolerance reported here are also powerful reminders of the critical need to develop improved treatment strategies for BU.

There is strong support for the role of public involvement in characterising the acceptability of clinical research [22]. We found that participants reported recognising the value of the research, particularly its role in testing candidate vaccines to prevent people from experiencing the ‘worst-case scenario’ in their community. Nevertheless, in the context of the risks identified, and again in the spirit of transparency, participants asked about alternative avenues for conducting similar research and had questions clarifying the value of some aspects of the research (for example, a vaccine’s usefulness in a low incidence disease). These discussions also suggested that the facilitators had established an open forum where participants felt empowered to question the motives and evidence provided by the investigators. Overall, the acceptability of the trial was largely dependent on the outcome. A large (i.e., uncontrolled) lesion was clearly unacceptable, and a small, curable lesion was overwhelmingly acceptable. The potential risks of the proposed study are therefore likely well within widely endorsed limits to acceptable risks to healthy volunteers in similar studies [23].

Focus group participants were also invited to discuss *who* may be interested in participating in MuCHIM, including perceived motivations to participate. Focus groups identified participant profiles reported in other CHIM research, including those who are altruistic and want to progress the field [19,24,25], those with a scientific curiosity or interest in biomedical research [24,26], and those who are ‘adventurous’ [26], although the motivation to describe the ‘story’ of their involvement, with a visible scar a ‘badge on their arm’ is a novel finding likely only relevant to skin CHIMs, such as this. A recurring suggestion was that those who live in or near an endemic area, or know someone personally impacted by the disease, may be motivated to participate in the model; this motivator has been described in malaria CHIMs [26]. Implementing this into the study’s design may be challenging, given participants are excluded if they reside within 2 km of a reported BU case in the community; alternative strategies to enable the inclusion of these participants will therefore need to be considered. Consistent with a study performed in the Netherlands [24], financial reimbursement was seen as important, but was not a strong theme in our analysis; this may not be generalisable to all settings.

Although there is a growing initiative to implement CHIMs in endemic settings [27], most CHIMs have been conducted in high income countries, often where the pathogen under investigation is not endemic [28]. This study explores a relatively novel setting of a neglected tropical disease endemic in a high-income setting. Our participants acknowledged that a MuCHIM may benefit people at risk of BU in low-resource settings such as West and Central Africa, where disease prevalence is greater. By including the voices of those with lived experience of BU, including public advocates, we hoped to avoid performing tokenistic research, a criticism sometimes levelled at investigator-initiated research where the public is expected to provide input on aspects with which they have limited direct experience and/or where study designs are not informed by the findings of public consultation [22].

It is impossible to identify every possible perception of the trial. Nevertheless, because individuals who work or study at the University of Melbourne are likely to live near a BU endemic region, this recruitment strategy was able to identify relevant stakeholders who will potentially have some personal or community benefit related to their contribution [29]. Most CHIM volunteers are typically higher education students [30], therefore the recruitment strategy aimed to involve a demographic likely to participate in MuCHIM. Although we did not record participant occupation at recruitment, each focus group did include at least one medical or biomedical research student, as reported during each meeting. As the target population likely to benefit from the research aligns with the population most likely to be recruited for MuCHIM (i.e., young adults in Melbourne undergoing tertiary education), the recruitment of focus group participants from this same population further reinforces the validity of the study setting. Any future applications of this MuCHIM in different demographic contexts will also benefit from further public consultation in that setting, as these findings may not be generalisable to other BU endemic communities (e.g., in West or Central Africa). Additionally, poor health ‘literacy’ may be perceived to undermine the potential for informed consent, because potential participants must demonstrate a comprehensive understanding of the risks and benefits of their involvement. This study therefore reinforces our plan to target recruitment of this participant profile.

Another strength of this study was the inclusion of the MuCHIM project’s principal investigator as a co-facilitator. As illustrated by previous CHIM focus group research [10], the concepts in CHIM trials require specialised content knowledge to fully respond to participant queries. The analytic method of reflexive thematic analysis was selected because it rejects positivist notions of researcher bias and embraces their experiences and perspectives as analytic tools, rather than labelling them as a threat [31]. Nevertheless, we recognise the risk of cognitive biases, particularly confirmation bias, in such research. It is for this reason that two facilitators, one fully independent of the MuCHIM project, were involved in all aspects of the analysis. SM and SS used a collaborative and iterative approach to the generation of themes, and themes were refined throughout a series of meetings and reporting stages (i.e., they were not fixed by any researcher). Nevertheless, we cannot exclude the possibility that participants felt obligated to provide desirable responses during the focus groups. At the conclusion of each focus group, participants were invited to send any supplementary comments or concerns directly to facilitators following the focus group meeting; however, no participant chose to do so. Additionally, both facilitators observed that participants appeared at ease in providing their feedback, even if they expressed concerns or differing viewpoints.

To progress MuCHIM further, we are now preparing participant information materials, using the principles described in this study, for review and refinement by stakeholders in a public forum. All participants of these focus groups will again be invited to participate. To refine the challenge protocol further, we have also submitted the protocol for scientific peer review in an open and transparent format [32], aligned with the themes emphasised as imperative to participants in the present study.

## Conclusions

This study presents the first description of public involvement in reviewing a proposed controlled human infection research design for BU in Melbourne, Australia. Based on the population studied, a MuCHIM would be acceptable to the public. The predominant theme generated from this research was the critical need for transparency in communication with potential participants. This communication must clearly outline the implications of participation, including the potential impact on their lives, the expected outcomes, and the ‘worst-case’ scenario of infection and scarring. While transparency is crucial, this study uniquely explored its nuanced application. Participants emphasised the importance of focusing on expected outcomes, while acknowledging the ‘worst-case scenario’ of infection and delayed diagnosis. To enhance informed consent, participants recommended including images and testimonials from individuals who have experienced BU. Furthermore, participants also sought clarity on the trial protocol’s rationale, such as challenge site selection. This information will guide the development of robust enrolment materials that effectively communicate risks and expectations to potential participants.

## Supporting information

S1 Material Abridged protocol reviewed by focus group participants. (DOCX)

S2 Material Focus group discussion guide. (DOCX)
