# Supplementary material for: Perceptions of a Buruli ulcer controlled human infection model: How, who, and why?
Source: PLoS Negl Trop Dis. 2025 Feb 5;19(2):e0012593. doi: 10.1371/journal.pntd.0012593 (PMC11819514; doi:10.1371/journal.pntd.0012593)
Supplement: S1 Material — (DOCX) [file pntd.0012593.s001.docx]

A human model of Buruli ulcer: Protocol for an initial *Mycobacterium ulcerans* controlled human infection study. **For focus group participants.**

*This summary should take around 60 – 90 minutes to review. Please note down any questions or issues you may want to discuss further during the focus group.*

# Background

*Mycobacterium ulcerans* is a slow-growing bacteria, causing the typically slow but progressive painless skin lesions known as ‘Buruli ulcer’ (BU), predominantly in Australia and Africa. BU is classified by the World Health Organisation as a neglected tropical disease, highlighting the importance of allocating resources to establishing improved preventative and treatment strategies. BU usually begins as a small red lump or patch of raised skin thickening (‘plaque’), which typically progresses over weeks to become a painless open wound (ulcer) that may become weepy. Rarely, a more painful and rapid BU may occur. In humans, it is not known to spread to internal organs, and spread to underlying bone is very rare, typically in people with large lesions and delayed diagnosis. Delayed diagnosis can lead to significant illness due to advanced ulceration, including deformity due to scarring, and high costs to the healthcare system. In Australia, cases continue to rise, and clusters have emerged in new locations. Antibiotics cure BU but the treatment course is long, and side effects are not uncommon, and reconstructive surgery may be required for severe lesions. Therefore, improved antibiotic regimens and preventative vaccines are important research priorities.

Although many candidate vaccines have been studied, several issues have prevented vaccine development. Studies have demonstrated at least short-term protection provided by the tuberculosis vaccine, bacillus Calmette–Guérin (BCG). Although earlier trials questioned the effectiveness of this vaccine in an African population, recent Australian research suggests a significant protective benefit from BCG vaccination, although routine use in Australia ceased in the mid-1980’s. BU in Australia tends to cause infection in small and unpredictable ‘hotspots’, which is a barrier to undertaking field trials to determine a vaccine’s effectiveness. For example, in the Bellarine Peninsula (Victoria) around 100,000 people would be required just to test the BCG vaccine. Combined with the slowly progressive nature of BU and its long incubation period (the time from exposure to clinical disease), a vaccine trial in the field would likely take decades and come at large cost.

A controlled human infection model (CHIM) of *Mycobacterium ulcerans* (‘MuCHIM’) is designed to deliberately cause BU in healthy adult volunteers. CHIMs have already successfully and safely been implemented for many infectious diseases. This research has the potential to advance our understanding of how human immune systems respond to the bacteria, and may be an efficient way to test vaccines and other treatments. For example, if everyone who is given the bacteria develops disease (i.e., 100% ‘attack rate’), then ‘MuCHIM’ could be used to test the BCG vaccine using just 28 participants. This approach could overcome the research bottleneck which limits vaccine testing and progression to larger clinical trials, mostly due to cost. Any number of candidate vaccines or new medications can be tested using this model, and any positive result would support a vaccination approach to curb the rise of BU in Australia. MuCHIM therefore has the potential to fast-track development of vaccines and new therapeutics for BU. Providing all reasonable measures are implemented to prioritise safety, there is ethical justification to establish such a model. By reducing the number of people infected with BU, many individuals visiting and living in these communities, and those in communities which may yet become endemic, may benefit from this research tool. It is unlikely that other approaches to testing BU vaccines in humans will be possible in any reasonable timeframe. Although *M. ulcerans* can infect a range of mammals, research animals, such as Guinea pigs, unfortuantely do not recreate the key features of human BU.

In brief, the trial will involve injection of the bacteria into the forearm, followed by monitoring of the participant until a lesion develops, which will likely take approximately 4 months. The lesion will then be treated with antibiotics, and excision (surgical removal of the lesion) will be an option. Blood tests will be taken throughout the study and the total trial duration for each participant will be around 12 months. Unlike BU in the community, participants will be rigorously followed up, ensuring very early diagnosis and treatment, so any issues are likely to be minimal compared to the real-world scenario.

Objectives and outcome measures

Overarching aim of the study (what’s the point?)

To establish a safe and acceptable *M. ulcerans* controlled human infection model of Buruli ulcer as a platform for accelerating development of preventive, therapeutic and diagnostic interventions, while understanding human immune responses to infection.

Study objectives (what do we want to do?)

1. Confirm safety and acceptability of the model in healthy adult participants
2. Establish a model with ≥ 60% ‘attack rate’ (at least 60% of people will develop BU)
3. Confirm the presence of BU bacteria (*M. ulcerans*) at the injection site if a lesion develops
4. Develop a model with ulcer cure rate of 100% after treatment (scarring may persist)

# Study design

MuCHIM is a controlled human infection study, involving the injection of a very low number of *M. ulcerans* bacteria into the subcutaneous tissue (the fatty layer under the skin) in healthy adult volunteers. Development of the MuCHIM will consist of two distinct stages. Stage 1 will include the establishment of policies and procedures required to optimise the safe and ethical conduct of the study. Stage 2 will incorporate the lessons and recommendations from Stage 1, leading to the first-in-human challenge of *M. ulcerans* in Stage 2A (the ‘pilot’ challenge stage).

Stage 1

- 1. Community consultation using focus groups (**this study**)
  2. Establishment of an independent safety review committee
  3. Creation of working cell banks of *M. ulcerans* for human infection
  4. Quality control testing of cell banks of the bacteria and regulatory approval
  5. Ethical and regulatory approval of the clinical trial

Stage 2

A. Recruitment of three participants for first-in-human infection (‘challenge’) with 10 – 20 bacteria in the side of the forearm (this can be considered a ‘pilot’ study)

B. Recruitment of 10 new participants for a dose confirmation study, using the lowest dose capable of infecting ≥ 2 of 3 participants in Stage 2A

Parameters for progression across Stage 2A/B include:

- Participants are able to tolerate study procedures
- No study-related serious adverse event (i.e., serious side effect)
- Confirmation of *M. ulcerans* in the lesion using standard diagnostic testing
- Resolution of infection in all participants (scarring may persist)

Parameters for progression to future vaccine/therapeutic trials:

- Above, plus establishment of a lesion in ≥ 60% of Stage 2A/B participants

# Recruitment and eligibility

# Participant recruitment

Following ethics approval, volunteers may be recruited using databases and/or advertising through posters, print, radio, or social media.

# Participant eligibility criteria

In Australia, most patients with BU are adults, therefore this study aims to recruit adults for study inclusion. In addition, BU disease severity and antibiotic complication rates are particularly problematic in children and the elderly. It is also generally considered unethical for children to be recruited into CHIMs. Therefore, initial studies will include adults aged 18 to 45 years old (inclusive). As the duration of participation is lengthy, only participants that are likely to comply for the study duration are eligible to participate. Measures to better facilitate follow-up will be prioritised, including questionnaires and digital photography of the challenge site using a portable electronic camera-enabled device (e.g., smart phone).

# Inclusion criteria (who will be eligible to participate?)

- Age between 18 and 45 years of age (inclusive) at the time of enrolment
- Capacity to provide written informed consent
- Willing and able to comply with all study requirements, including antibiotics
- Planned residence near study site (≤ 2 hr drive or public transit) for at least 12 months from enrolment
- English language proficiency (to ensure comprehensive understanding of the study and their proposed involvement)
- Individuals with childbearing potential with a negative urine pregnancy test at screening and willing to practice acceptable contraception until 30 days after antibiotic completion (see Table 1)
- Provides written consent to discuss medical history, and to share correspondence, with their nominated general practitioner or other relevant health care provider
- Up-to-date with tetanus vaccination, or willing to receive vaccination prior to challenge

# Temporary exclusion criteria (enrolment into the trial needs to be delayed)

- Use of any antibiotic within 28 days of subcutaneous challenge
- Any vaccination within 28 days of subcutaneous challenge
- Fever or other transient medical illness (e.g., cold/flu)

# Exclusion criteria (who will **not** be eligible to participate?)

- Clinically significant history of skin disorder, malignancy (cancer), cardiovascular disease, respiratory disease, gastrointestinal disease, liver disease, kidney disease, endocrine (hormonal) disorder, haematological (blood) disease or neurological disease^*^
- Clinically significant psychiatric disorder anticipated to interrupt follow-up^*^
- Body mass index ≥ 25 kg/m^2^ (i.e., overweight or obese)
- Primary or secondary immunocompromise (i.e., weakened immune system due to a genetic condition, or acquired due to chemotherapy or other treatment), based on history, examination and/or investigations
- Current or recent (within 3 months) habitual (i.e., regular) smoking, including cigarettes, cigars, e-cigarettes, vaping, or smoking of recreational drugs
- History of sustained hazardous alcohol consumption, defined as ≥ 10 standard drinks per week in the last 12 months
- Unwilling or unable to abstain from alcohol during antibiotic treatment
- Medication or other interaction with antibiotics used to treat BU
- History of allergy to any of the antibiotics used to treat BU
- History of allergy to local anaesthetic
- History of allergy to corticosteroids (a medication occasionally used to treat BU)
- Abnormal baseline electrocardiogram (ECG) findings (i.e., heart electrical rhythm)
- History of hearing impairment or abnormal baseline hearing test
- For individuals of childbearing potential:
  - Current or planned pregnancy
  - Current or planned breastfeeding
  - Unwilling or unable to use acceptable contraception (Table 1) from time of challenge until 30 days after antibiotic completion
- History of poor wound healing or excessive scarring
- History of allergy to any ingredient in the challenge dose
- Previous or current Buruli ulcer, tuberculosis or leprosy
- Previous challenge with *M. ulcerans* JKD8049
- Resides in close proximity to endemic area (within 2 km) based on Victorian Department of Health epidemiologic data
- Family member/co-habiting with someone with a history of BU
- Previous history or examination finding consistent with *M. bovis* BCG vaccination
- Latent (dormant) tuberculosis, chronic hepatitis B or hepatitis C (based on their medical history or screening tests)
- Vision impairment preventing self-examination of challenge site (and/or unable to use alternative to soft contact lenses, as these are stained by a key antibiotic to treat BU)
- Unable to tolerate injections
- Enrolment in another study at the same time which uses an investigational product or collects volunteer’s blood
- Any major issue with accessing participants’ veins for blood tests
- Any condition, including medical and psychiatric conditions that in the opinion of the Investigator, might interfere with the safety of the volunteer and/or study objectives

^*^ Clinical significance is at the discretion of the Study Investigator.

# Schedule of events and procedures

The trial will be divided into four distinct periods: *screening, challenge, treatment,* and *healing.*

# 1. Screening period

Informed consent

Written informed consent will be required prior to participation. Prospective participants will be invited to discuss the study during a facilitated meeting, which includes a brief presentation, which may occur as a group discussion with study investigators. The risks of the trial will be described, and participants will also be allowed to ask questions privately. They may consider their involvement for up to 28 days, to allow them adequate time to consider participation.

To assess their capacity to provide consent, participants will be invited to complete a multiple-choice quiz to demonstrate their understanding of the study and to ensure researchers have communicated details of the study appropriately. Incorrect answers will be explained by the researcher, and participants will have the opportunity to repeat the quiz again. If the study team are satisfied that the participant is voluntarily offering to participate in the trial, they will be invited to provide written informed consent. Written informed consent will be obtained by the responsible clinician on the day of any further procedure.

# Screening procedure

Screening aims to select participants at low risk of disease or treatment related complications. During screening, a medically qualified researcher will check that the prospective participant meets all eligibility criteria. Participants found to have any exclusion criteria on history or examination will not go on to have investigations performed. Should a previously unrecognised condition be identified during screening, the participant will be informed by a qualified medical practitioner, and referred to their general practitioner (GP) or specialist for further investigation and management as relevant.

### Medical history

The initial clinic visit will include a detailed medical history to ensure that participants are healthy and at low risk of complications, including obtaining any concurrent medical conditions, medications (including non-prescription and recreational drug use), smoking history, alcohol consumption, allergies, and vaccination history. History will also include a discussion on pregnancy and pregnancy planning, and the ability to use acceptable methods of contraception.

### Physical examination

The initial clinic visit will include a targeted clinical examination including recording vital signs, weight and height to calculate body-mass index (BMI). Criteria will exclude volunteers who are overweight/obese, which is a risk factor for failure of BU treatment. A skin check will document Fitzpatrick skin type (i.e., the fairness of their skin tone), as darker skin tones are associated with increased scarring risk. The researcher will inspect for any evidence of previous or current BU or BCG vaccination (usually a small scar on the outside of the shoulder/upper arm). A thorough physical examination will be performed to evaluate for previously unrecognised medical conditions.

Fitzpatrick skin type

Participants with a Fitzpatrick skin phototype ≥ 5 (i.e., darker skin tones) are at higher risk of scarring. Nevertheless, their inclusion has important implications for understanding BU in people of diverse backgrounds. They will therefore require an additional element of informed consent to participate, bearing this additional increased risk in mind.

### Investigations

Volunteers will be screened for immunodeficiency (weakened immune system); including diabetes, which is a known risk factor for BU, the more severe oedematous (swollen) lesions, and may impair wound healing. Other investigations will include blood testing for viruses (such as HIV), and screening for low white blood cells and low antibody levels. Infections that may increase the risk of liver inflammation (hepatitis B and C viruses) will also be tested. Screening for tuberculosis with a blood test will be performed. Investigations will also target potential issues related to antibiotics, including abnormal baseline ECG (heart rhythm), electrolyte and kidney disorders, hearing impairment, and pre-existing liver disease.

# Sampling

Sampling throughout the trial will include blood collection (maximum 450 mL during any 3 month period) for routine testing (electrolytes, kidney and liver function, and measures of inflammation) and full blood analyses, in addition to tests which will characterise the person’s immunological response to infection. Blood will be collected at the prespecified times described under ‘Study procedure’ during the trial.

|  |  |
| --- | --- |

### Response to excipients (ingredients in injection) and monitoring

After signed consent is obtained and all eligibility criteria are met, the participant will be monitored as an outpatient (day-stay) to enable a ‘sham’ challenge in the opposite forearm to that used in the actual challenge. This will test their response to the preservatives and chemicals in the media without the bacteria, although no ingredient is known to be harmful. It also establishes if scarring occurs due to the injection itself, and that no other local skin reaction develops. They will be observed for 4 hours, with frequent observations. The ‘sham’ challenge will be performed in the same setting as the subsequent challenge (see ‘Study setting’). Participants will be required to record a virtual diary using a secure online platform (‘RedCAP’) throughout the study, and participants can upload a photograph of the challenge site to this platform. They will be asked to photograph the ‘sham’ challenge site daily for 3 days, and the site will be examined at each subsequent face-to-face visit. Participants will be instructed to hold the camera 15 – 20 cm from the challenge site, in a well-lit environment, using flash if available. Participants will be provided with a paper tape measure to record the size of any lesion or reaction. Questionnaires will evaluate symptoms and tolerability of procedures using a few simple questions in their participant diary.

# 2. Challenge period

# Study setting

This single centre study will be conducted at Doherty Clinical Trials (DCT) in East Melbourne, Victoria. This facility was established to facilitate the establishment of human challenge trials, and is supported by clinicians with experience in this field of research. The centre includes dedicated inpatient and outpatient clinical care areas, a pharmaceutical preparation area and access to qualified medical personnel. The ‘challenge’ (i.e., injection of bacteria into the skin) will be performed in a dedicated space within the trial facility, with personal protective equipment observing ‘contact’ precautions, including protective eyewear in case of accidental splash. Participants will be monitored as outpatients for 4 hours after challenge. Due to the long period before infection develops (4 – 5 months in Victoria, Australia, maximum 9 months), and the long duration required for follow-up, all participants will be followed up as outpatients at the DCT centre.

Challenge strain manufacture and cell banking

The proposed challenge agent, *M. ulcerans* JKD8049, has been extensively characterised for the purposes of human challenge. It is a fully antibiotic-susceptible, non-genetically modified Australian isolate, collected from a middle-aged male with a typical BU acquired in Point Lonsdale, Victoria. Quality control testing of 10% of the vials will be performed to ensure there is no contamination and to confirm the dose.

# Dosing of challenge strain

The dose of bacteria required to infect humans is likely to be very low. First-in-human challenge will begin with a dose of 10 – 20 bacteria, as doses in this range have previously demonstrated an attack rate of 100% in research mice. Recruitment of additional participants for dose-escalation (increasing doses) will not occur for at least 9 months after this dose fails to cause infection, as this is the maximum reported incubation period. If needed, dose-escalation will increase the number of bacteria received per participant by 20. Each increment will challenge three participants (stage 2A), and if ≥ 2 of 3 are successfully challenged, then this dose will be used to challenge 10 subsequent volunteers in a dose confirmation study (stage 2B). New participants will always be recruited for dose confirmation studies.

# Challenge site

The inner aspect of the forearm (little finger side), approximately one-third of the distance between the elbow crease and the wrist, is the selected site. This site is preferred as it:

1. Is a common site involved during natural infection

2. Is not associated with an increased risk of developing a severe swollen lesion

3. Minimises the risk of spread to bones and joints, which are already very rare

4. Minimises the possibility of muscles/tendons/skin stiffness developing due to scarring

5. Is simple to remove with minor surgery with closure of wound (i.e., with stitches)

6. Is expected to be less visible to participants if scarring develops

7. Can be easily self-examined and photographed by participants

The arm will be measured from the elbow crease to the wrist, and the challenge site will be estimated using this distance (see Fig. 1). The non-dominant arm will be suggested, as it may be easier to self-inspect and to do dressing changes, although the side can be nominated by the participant.


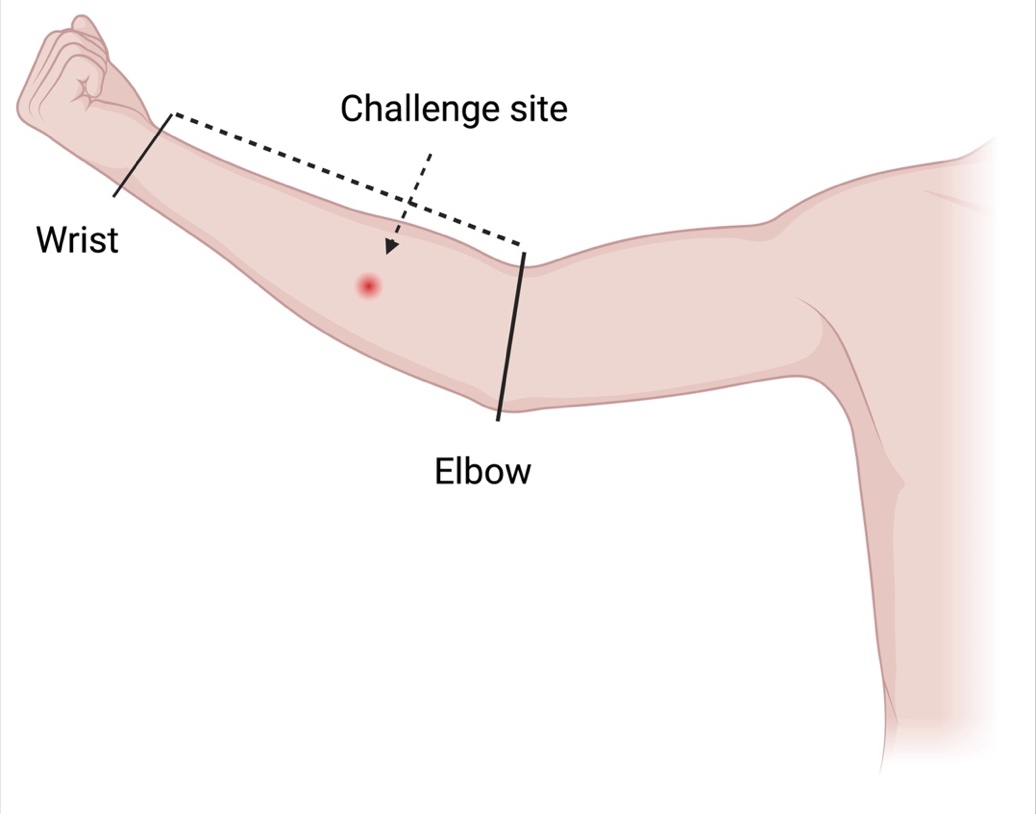


**Figure. 1**: The proposed challenge site will be along the medial (inner) aspect of the forearm, one-third of the distance from the elbow to the wrist. Created with BioRender.com.

# Administration

If required, the injection site will be prepared by clipping the hair around the site to allow the dressing to attach and improved visualisation. The skin will be disinfected with a 70% alcohol wipe and allowed to air dry for 30 seconds. After thawing the preserved cells, the vial will be diluted to the required dose and injected in a maximum volume of 0.1 mL by trained study staff using a sterile, small, thin needle, at a depth of ~ 2 mm (for comparison, 0.5 mL is typically injected in most vaccines). The skin may be ‘pinched’ to aid injection. The bacteria will then be injected slowly over ~ 10 seconds. Following injection, a cotton swab will be used to apply gentle pressure over the injection site as the needle is withdrawn, to minimise backflow of the liquid. Simple bandaging without antiseptic will be used to cover the injection site. Simple pain relief (e.g., paracetamol) will be offered if needed. Prior to discharge, participants will be instructed on the possible lesion appearance, with take-home visual instructions and images. Participants will also be instructed on how to photograph the challenge site and how to navigate the online portal to upload images and complete the virtual diary.

# Monitoring after challenge

After challenge, participants will complete their participant diary daily for 3 days, including photographs of the challenge site. Thereafter, virtual monitoring during this period will include twice weekly participant diary entry. In-person review for physical examination and blood testing will occur 3, 7 and 14 days after challenge, and monthly thereafter. Participants will otherwise be asked to examine the challenge site daily to monitor for any lesion. If any visible lesion develops, they will be instructed to promptly notify a trial researcher. The development of a lesion indicates the end of the ‘challenge’ period.

# 3. Diagnosis and treatment periods

# Case definition

The appearance of a nodule (lump under the skin), plaque (flat, firm area of raised skin), papule (similar to a pimple), localised induration (a patch of lumpy or thickened raised skin), erythema (redness), generalised oedema (swelling) or ulceration, at or near the challenge site, will be classified as a ‘probable’ case. A swab or biopsy of the lesion will be used to ‘confirm’ the lesion as a BU. The ‘PCR’ test for BU is the current ‘gold standard’ diagnostic tool.

# Expected outcome (result)

If a participant reports a lesion, they will be reviewed by the research team within 48 – 72 hours. The expected outcome is that an ‘early lesion’ (patch of redness and/or swelling) will develop into a ‘pre-ulcerative lesion’ (nodule/plaque/papule). In the event that they develop an ‘early lesion’ or ‘pre-ulcerative lesion’, the participant will be asked to monitor the lesion and return for review if any ulceration occurs. If any ‘early lesion’ fails to progress into a ‘pre-ulcerative’ lesion despite 10 days of monitoring, treatment will be provided. If a ‘pre-ulcerative’ lesion persists after 7 days without progressing to ulceration, treatment will be provided. Face-to-face follow-up will be weekly for 4 weeks after any lesion is reported.

# Antibiotic treatment

The internationally recommended antibiotic regimen is oral rifampicin (standard adult dose 600 mg, once daily) and clarithromycin (standard adult dose 500 mg, twice daily) for 8 weeks, which results in very high cure rates. Relapse risk (~1%) (i.e., recurrence after treatment) will be further minimised by selecting volunteers without risk factors for relapse, which include immunocompromised people and people who are overweight/obese. In the very unlikely event that participants relapse, a repeat course of antibiotics, typically rifampicin with an alternative antibiotic (e.g., ciprofloxacin), with or without surgical removal of the lesion, remains standard of care. In Australia, research evidence suggests that 6 weeks of antibiotic therapy is likely to be as effective as 8 weeks of therapy in select patients with small ulcers, with 100% of small lesions successfully treated with 6 weeks of antibiotic therapy. Participants will be prescribed antibiotic treatment according to the schedule listed in ‘Study procedure.’ They will be provided with an information pamphlet on side effects, when and how frequently to take the medication, and will be asked to report all side effects via their participant diary (see ‘Risk assessment’ for further detail). A dosette box with each day of the week clearly labelled will be provided to support participants with the antibiotic schedule.

Wound care

All lesions/wounds will be reviewed by an experienced clinician to ensure appropriate dressings are applied and the frequency of dressing changes is optimised (typically every second day, depending on how much discharge is being produced from the wound, if any). Participants should be able to manage their own dressing changes, after being provided with an ample supply of dressing equipment and training on aseptic technique (to minimise wound contamination). Written instructions and telephone contact details of study investigators will be provided to participants in case of wound deterioration. Another bacterial infection of the BU lesion is rare but should be considered if the wound becomes painful and/or acutely inflamed. In this event, a clinician will evaluate the participant and if deemed appropriate, will prescribe further antibiotic treatment that has minimal risk of interacting or exacerbating side effects from the BU treatment antibiotics.

All wounds healing without surgery will be dressed appropriately with an absorbent dressing. For open wounds, over-the-counter topical preparations (gels/ointments) such as ‘Flaminal’ may be used; these allow the base of the wound to remain hydrated, while chemicals in the ointment debride (remove) unhealthy tissue, and contain antibacterial properties to prevent secondary bacterial infection. Participants will also be instructed to continue their regular activities, but ideally minimise injuring the wound, as this may exacerbate inflammation and wound breakdown. To minimise scarring, participants will be provided with a hypoallergenic moisturiser (with sun protection) to aid scar healing.

# Surgical treatment

Surgery is not usually required in the treatment of BU but still has a role in shortening the duration of antibiotic treatment. In this trial, participants will be given the option to have the lesion removed by a plastic surgeon. This would involve injection of local anaesthetic around the lesion, removal of the lesion, followed by stitches. The procedure is similar to having a mole removed. Lesion excision reduces the duration of antibiotics required. Australian research suggests that 14 – 28 days of antibiotic therapy is adequate to cure those who receive antibiotics combined with surgery. We propose using a duration of 4 weeks if the excised tissue margins (edges) are involved (i.e., if there is microscopic evidence of inflammation and/or bacteria at the edge of the removed skin), and 2 – 4 weeks if the tissue margins are clear, guided by the patients’ ability to tolerate antibiotics (i.e., if the participant has issues with antibiotics, then 2 weeks of treatment may be acceptable). The duration of 4 weeks if margins are involved is based on research suggesting that bacteria are killed in tissue following 28 days of treatment in mice and humans. Participants who cannot tolerate antibiotics (at any stage of treatment) will also have the option of surgical excision. Surgical excision is anticipated to leave a thin scar. Surgery will be performed by an experienced plastic surgeon under local anaesthetic.

# Lesion sampling

In the case of an ulcerated lesion, a swab will confirm the presence of *M. ulcerans* DNA within the lesion. For non-ulcerated lesions (e.g., nodule), a minimally-invasive biopsy device will be used to test the presence of *M. ulcerans* DNA. This biopsy is not expected to leave a scar, as the wound created is just 0.21 mm in diameter. In addition, a 3 mm punch biopsy (which may cause a scar) will also be performed to provide a comparison to the minimally-invasive test (i.e., the minimally-invasive test may be used in future trials without the punch biopsy if it able to confirm the BU)*.* A single Steri-Strip will be used to bring the punch biopsy edges together. For participants who elect to have the lesion removed after the confirmatory test, the tissue removed will be processed for additional immunological testing for research purposes. All participants who do not undergo a surgical excision will be invited to have an additional 4 mm punch biopsy collected at the time of diagnostic sampling (see ‘Exploratory analyses’).

# Monitoring

Once antibiotic treatment is started, a researcher will check for side effects with weekly ‘adverse reaction’ screening (in person or telephone call), and a qualified doctor will review the participant if anything is reported. 'Safety’ blood tests will be performed at baseline (before antibiotics) and weekly for 4 weeks, then 2-weekly thereafter for a further 8 weeks. ECG will also be performed before antibiotics and 1 – 2 weeks into antibiotic therapy. Participants will also complete their participant diary twice weekly, including reporting of any antibiotic side effects. Regular face-to-face outpatient follow-up will enable prompt clinical evaluation, initiation of treatment and wound care as required. For an exploratory analysis, participants may also be invited to provide a faecal sample (using a self-collection kit) for microbiome analysis prior to, during and after the completion of antibiotic therapy.

After a lesion is noted, participants will be asked to complete the Dermatology Life Quality Index (DLQI) questionnaire, which is used to measure the impact of skin disease on their quality of life. Participants will be invited to do this questionnaire every week for 1 month after the lesion is first reported, then monthly until study completion. The Generalised Anxiety Disorder 7 questionnaire (GAD-7) will also be used to measure their mood, beginning at the time of challenge and continuing monthly until a lesion is reported; the questionnaire will then be performed at the same intervals as the DLQI.

Schedule of visits and procedures

**1. Screening period**


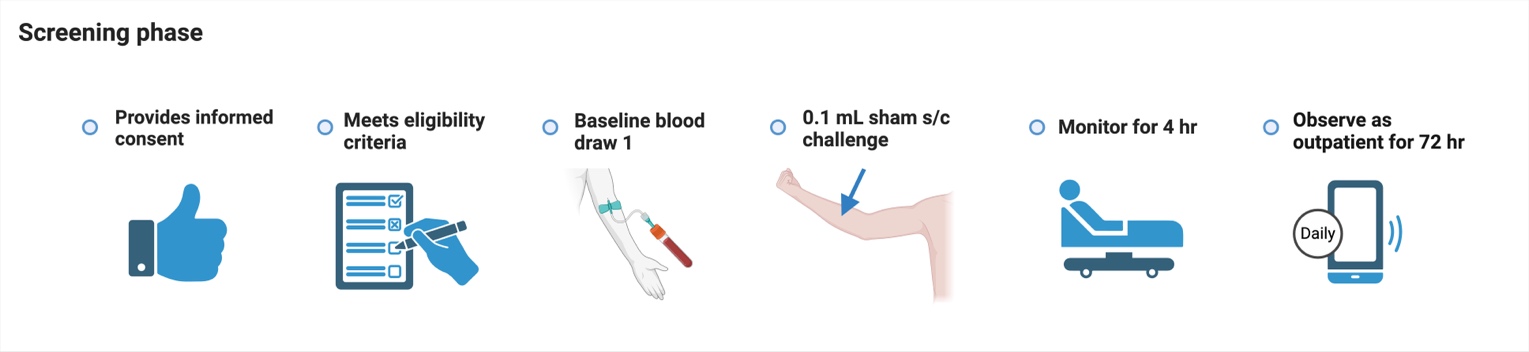


Participants will not proceed to JKD8049 challenge if they develop any serious adverse reaction. The telephone icon represents their virtual participant diary, including self-collected photograph. s/c: subcutaneous. Created with BioRender.com.

**2. Challenge period**


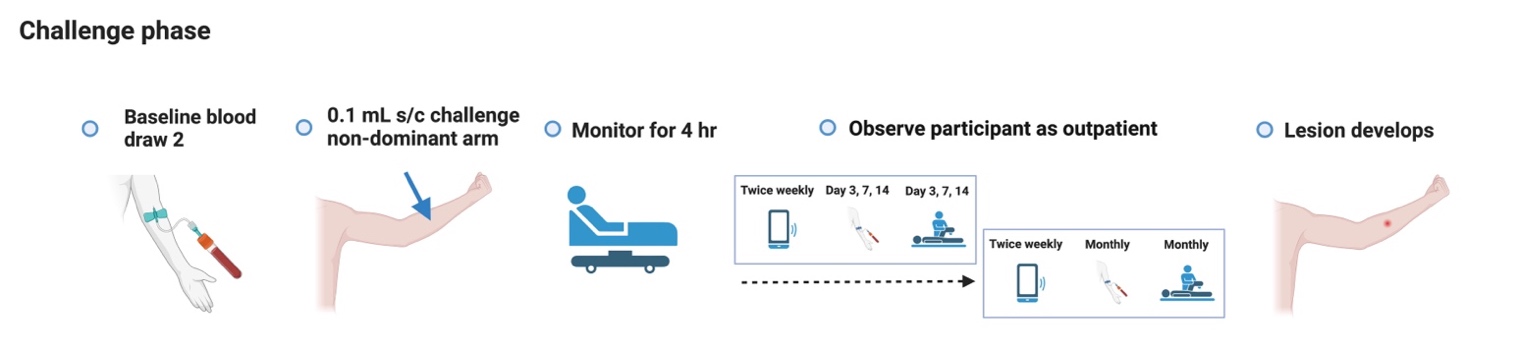


The icon of the light blue figure examining the dark blue figure represents a face-to-face visit, including physical examination. Created with BioRender.com.

**3.1. Treatment** – Expected outcome 1A: Surgical excision of early lesion


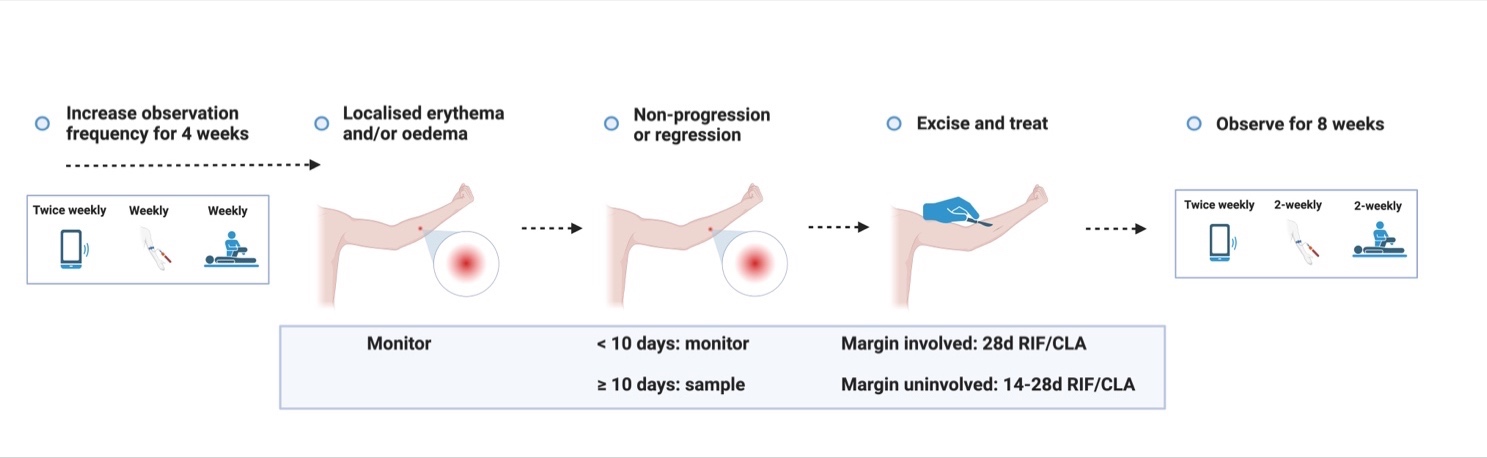


If a local area of redness (erythema) +/- oedema (swelling) fails to progress (or regresses) after 10 days of close observation, it will be treated surgically, with the duration of antibiotics post-operatively dependant on whether the margin was involved. RIF/CLA = rifampicin and clarithromycin antibiotics. Created with BioRender.com.

**3.2. Treatment** – Expected outcome 1B: Surgical excision of pre-ulcerative lesion


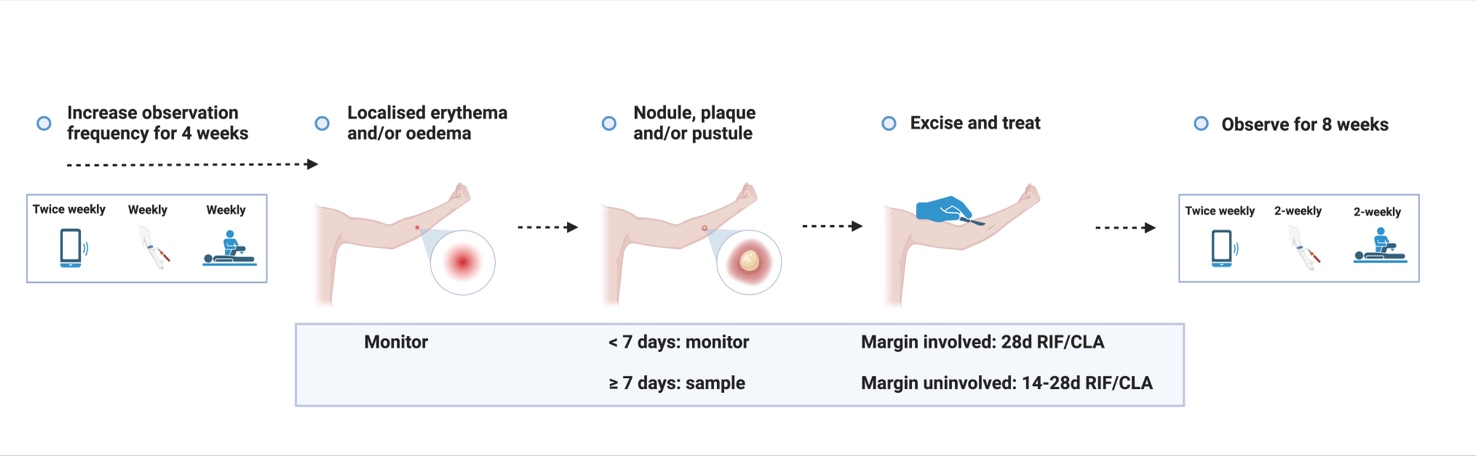


If a local area of redness (erythema) +/- oedema (swelling) progresses into a nodule (lump), plaque (flat, firm area of skin) or papule / pustule (similar to a pimple), it will be monitored closely for up to 7 days. If one of these lesions has been present for 7 days or more, it will be treated surgically with the duration of antibiotics post-operatively dependant on whether the margin is involved. RIF/CLA = rifampicin and clarithromycin antibiotics. Created with BioRender.com.

**3.3. Treatment** – Expected outcome 1C: Surgical excision of ulcer


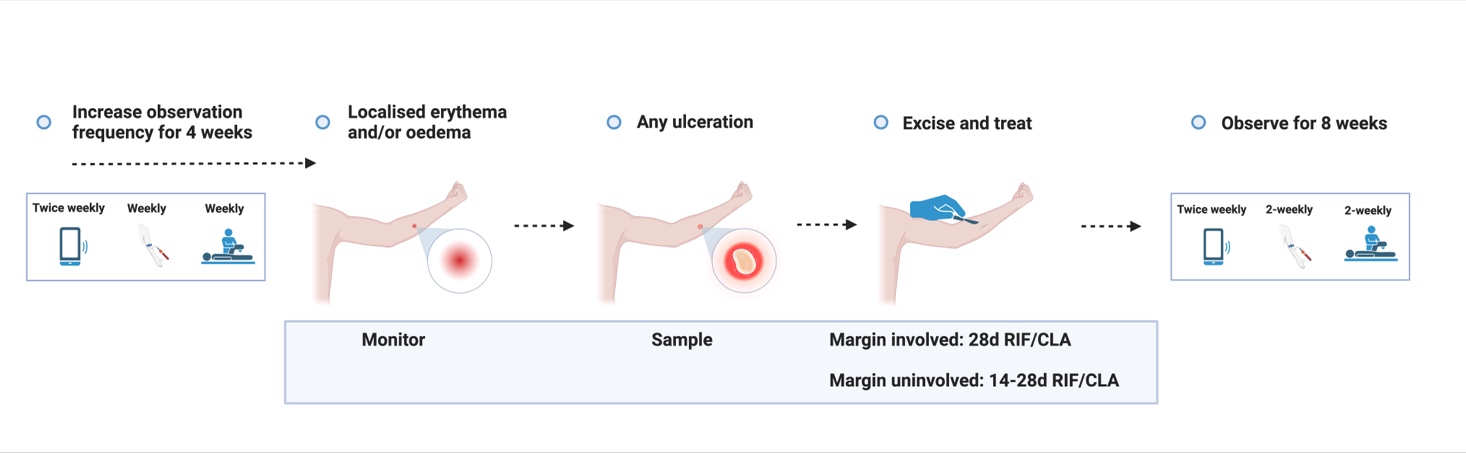


If a lesion becomes an ulcer at any stage, it will be treated surgically with the duration of antibiotics post-operatively dependant on whether the margin is involved. RIF/CLA = rifampicin and clarithromycin antibiotics. Created with BioRender.com.

**3.4. Treatment** – Expected outcome 2A: Antibiotic treatment without surgery


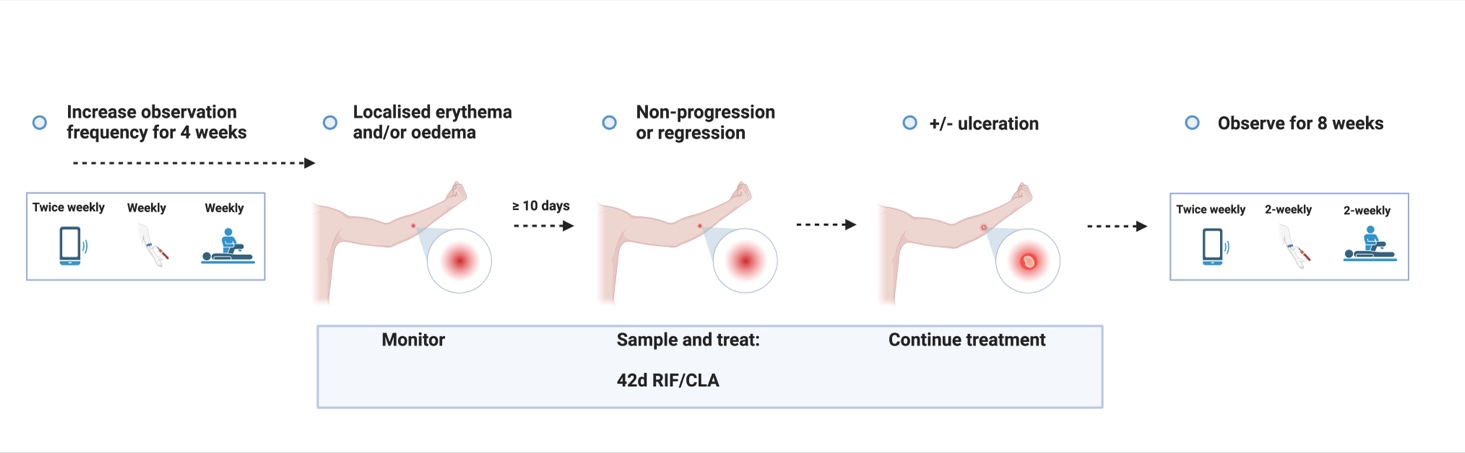


If a local area of redness (erythema) +/- oedema (swelling) fails to progress (or regresses) after 10 days of close observation, it will be treated with antibiotics for a total duration of 6 weeks. If ulceration develops, this will not alter the planned duration of treatment. RIF/CLA = rifampicin and clarithromycin antibiotics. Created with BioRender.com.

**3.5. Treatment** – Expected outcome 2B: Antibiotic treatment without surgery


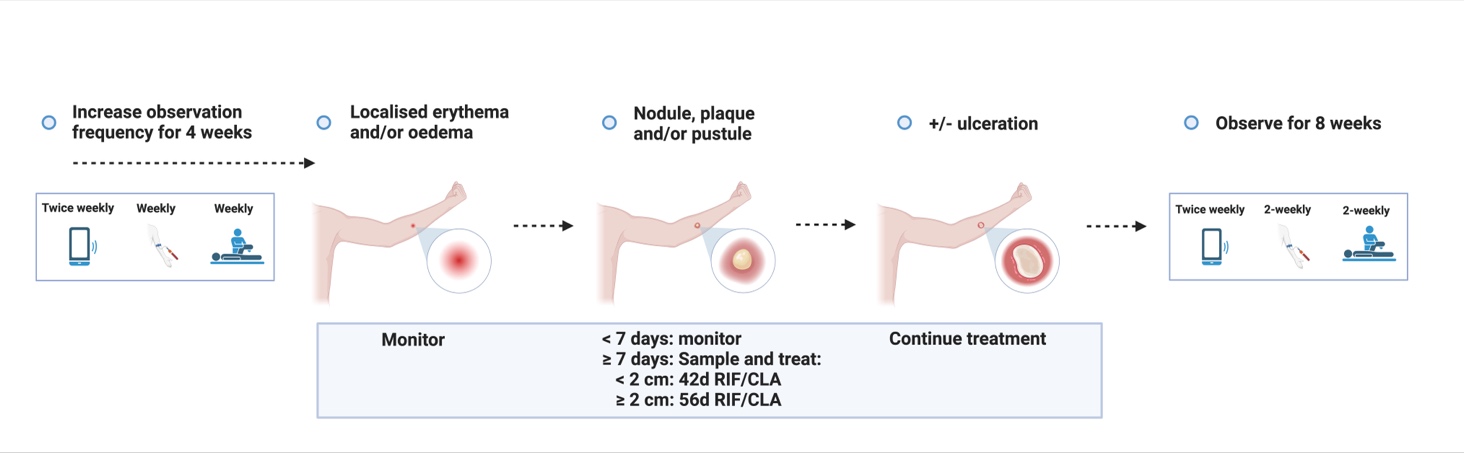


If a local area of redness (erythema) +/- oedema (swelling) progresses into a nodule (lump), plaque (flat, firm area of skin) or papule / pustule (similar to a pimple), it will be monitored closely for up to 7 days. If one of these lesions has been present for 7 days or more, it will be treated with antibiotics. The duration of antibiotics will depend on the maximum size of the lesion (6 weeks if it is less than 2 cm in maximum diameter or 8 weeks otherwise). RIF/CLA = rifampicin and clarithromycin antibiotics. Created with BioRender.com.

**3.6. Treatment** – Alternative outcome: No lesion at 9 months


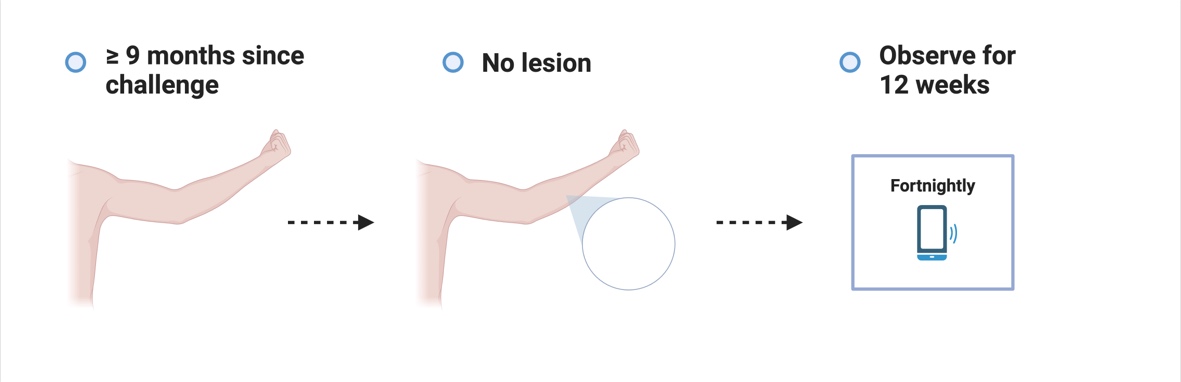


These participants will no longer be eligible to participate in a subsequent dose escalation study. They will all be asked to contact trial researchers in the unlikely event that a lesion develops after study completion. Created with BioRender.com.

**3.7. Treatment** – Unexpected outcome: No lesion, participants exits trial prematurely


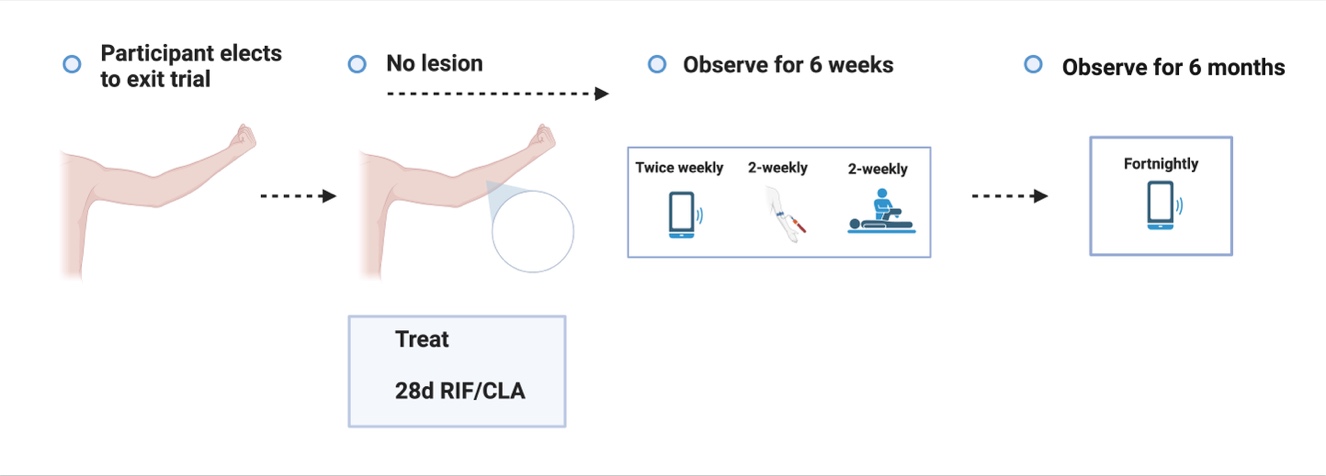


Participants who meet the STOP criteria will be offered pre-emptive treatment, and a follow up period of 6 weeks will be offered. The participant will be followed up using the least restrictive method thereafter if the above plan is unable to be observed (e.g., telephone, email) and will be linked in with their usual GP. RIF/CLA = rifampicin and clarithromycin antibiotics. Created with BioRender.com.

**3.8. Treatment** – Unlikely (adverse) outcome: Cellulitic / oedematous lesion


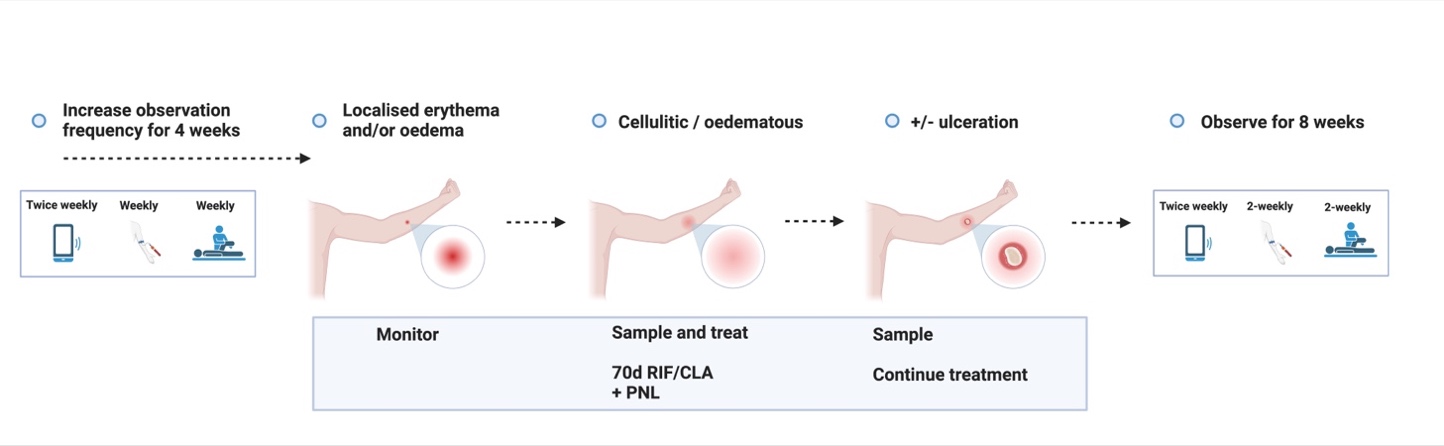


In this study, cellulitic/oedematous lesions are defined as erythema (redness) and/or oedema (swelling) ≥ 5 cm (in maximum diameter) at the challenge site ≤ 7 days from when the lesion is first reported. Urgent clinician review (within 24 hours) will also evaluate and consider treatment of superimposed non-*M. ulcerans* skin/soft tissue infection. RIF/CLA = rifampicin and clarithromycin antibiotics, PNL = prednisolone. Created with BioRender.com.

**4.1. Healing period** – Expected outcome


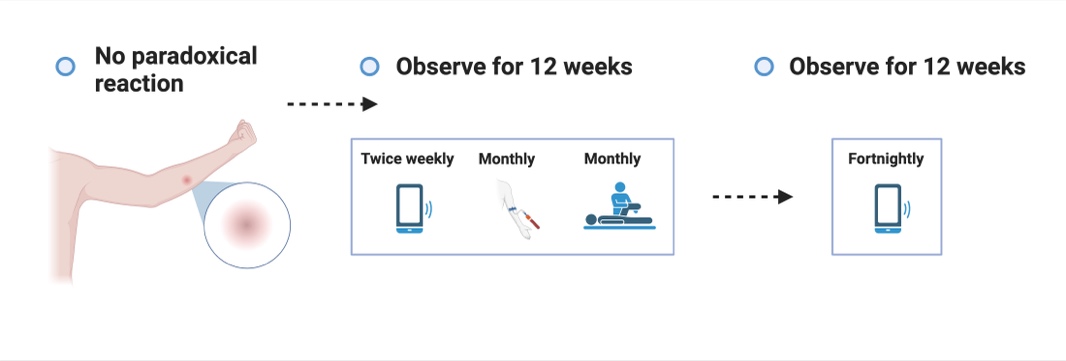


Participants who heal as expected will be followed up for 12 weeks with a monthly blood test and face-to-face visit, then via their virtual diary alone for a further 12 weeks before completing the trial. Created with BioRender.com.

**4.2. Healing period** – Unlikely outcome: Paradoxical reaction


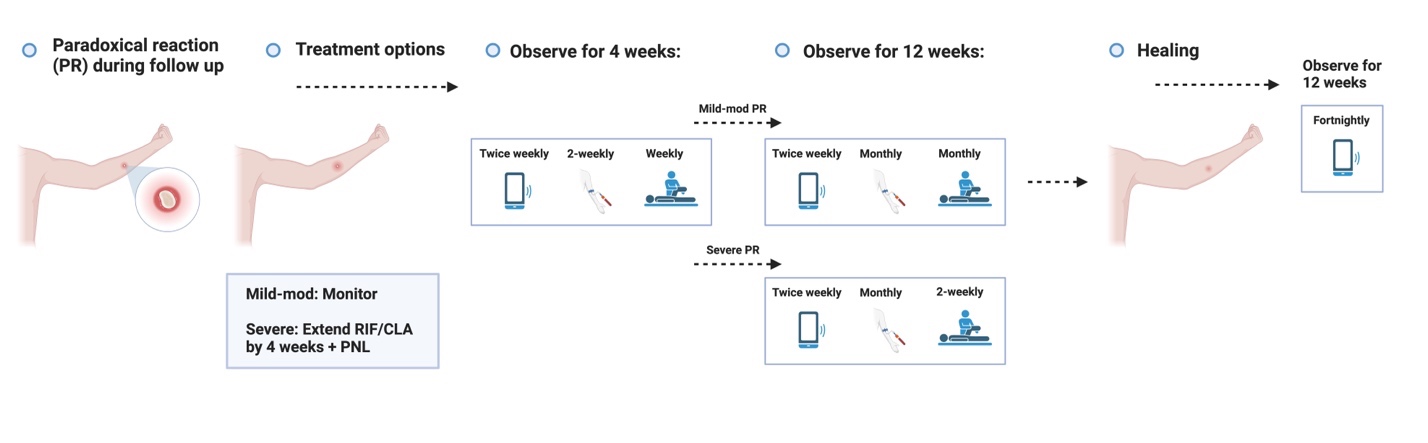


Participants who develop a paradoxical reaction (unexpected worsening of the lesion while on treatment) will be followed up weekly for 4 weeks. If the paradoxical reaction is mild or moderate, it will be monitored without any intervention. If it is severe, antibiotic treatment may be extended and prednisolone (PNL) may be required to reduce the inflammation. After this 4 week duration, the follow-up will reduce to 2-weekly for those who had a severe paradoxical reaction or monthly for those who had a mild or moderated paradoxical reaction. Created with BioRender.com.

# STOP criteria

For participants who are already enrolled and have been challenged with *M. ulcerans* JKD8049, the STOP criteria may be implemented, with the approval of the study steering and safety committees. Participants who meet the STOP criteria will be offered pre-emptive antibiotic treatment and follow-up will continue as per ‘Study Procedure’, with participants encouraged to participate in all safety interventions (or if not possible, the participant will be linked into care with their GP, with additional phone or email follow-up, if feasible):

- Medical illness: STOP if they cannot continue to be involved in the trial due to a medical condition that arises during the course of follow-up and may result in poorer outcomes if allowed to progress
- Engagement: STOP if the trial team have concerns about the participant’s ability to commit to safety checks and frequent communication.
- Study is discontinued for any other reason.

### 4. Healing period

The beginning of this period is defined as 12 weeks after a lesion is first noted, although healing of the lesion is expected to begin at some stage during antibiotic therapy. This period is anticipated to include ongoing wound healing after the completion of antibiotics and scar maturation. In-person monitoring during this period will occur monthly for 12 weeks, including blood tests, and twice weekly participant diary entry. After the final face-to-face visit, the patient diary alone will be used for routine follow-up for a further 12 weeks.

# End of study

Stage 2A of the study will end if at least two participants are successfully challenged and when all participants complete their final study visit. Each participant’s final in-person visit is anticipated to occur 9 – 10 months after recruitment (with an additional 12 weeks of infrequent virtual follow-up to document scar maturation). This assumes an incubation period of approximately 3 – 4 months, as lesions are likely to be noted by participants sooner than may otherwise be reported in the field. Participants who have completed the trial will be provided with the contact details of medical clinics with experience managing BU, as well as the trial management team, in the unlikely event that they develop a lesion after the end of the study. At study completion, all participants will complete an exit questionnaire, which will also inform future applications of the study.

# Additional research samples (‘exploratory analyses’)

# Understanding changes to the ‘microbiome’ during the study

This optional study aims to understand the impact of antibiotics on normal skin and gut bacteria (‘microbiome’) over time. If participants consent, skin swabs will be collected at specified times during usual face-to-face visits, and faecal microbiome samples may be collected using a dedicated self-collection kit during the study.

# Immune responses in the skin

For participants who prefer to have the lesion surgically removed, any skin tissue surgically excised will offer a rich source of additional information about immune responses to infection in the skin. For participants who prefer to have any lesion treated with antibiotics alone, they will have the option to have an additional 4 mm punch biopsy performed at the time of the diagnostic biopsy (or at the time of diagnostic swab in the case of ulcerated lesions).

# Risk assessment

CHIMs can generally only be established in treatable diseases where permanent disease is extremely unlikely. The following section describes the key risks of the trial.

# Antibiotics

A unique aspect of this trial is that the risk of antibiotic adverse events may be greater than the risks related to a small, early BU. Nevertheless, the risks related to antibiotic use are well characterised, and these antibiotics are prescribed by clinicians for many other conditions. Australian evidence suggests that antibiotic complications are not uncommon, although risk factors for these side effects are well described, such as poor kidney function, which again highlights the importance of careful participant selection. Rare side effects of all treatments include allergic reactions, which are minimised by ensuring participants have not had allergic reactions to these, or similar medications, in the past. Common but short-lived side effects from rifampicin and clarithromycin include nausea and reduced appetite, and dysgeusia (altered taste sensation) due to clarithromycin. Diarrhoea may also occur. Patients will be encouraged to maintain adequate hydration, and anti-nausea tablets may be prescribed. Dividing rifampicin into two daily doses (instead of once daily) may also improve symptoms of nausea. If diarrhoea is associated with severe symptoms (persists > 48 hours, abdominal pain, fever) then the participant will be reviewed for another potential complication, *C. difficile* diarrhoea, which is a bacterial gut infection which occurs after antibiotic use; *C. difficile* is treated with another oral antibiotic which is typically taken for 10 days.

Red discoloration of bodily fluids is commonly observed when taking rifampicin, this is benign and resolves after treatment, but it may cause alarm if participants are not warned in advance. Participants who use soft contact lenses should consider alternatives due to potential staining. Due to the risk of drug interactions with any trial antibiotic, participants will be instructed to inform the trial team of any new medications or non-prescription therapies.

Blood testing may show mild transient liver-related blood test abnormalities, although important toxicity to the liver is rare; it is not currently routine practice to monitor liver function in patients with BU on these antibiotics without an identified high risk of liver injury, but for this trial, liver-related blood tests will be monitored during treatment as an additional precaution. Based on a previous large trial of people taking rifampicin for 4 months, liver toxicity due to rifampicin occurs in 0.3% of people, with allergic reactions (0.2%) and haematological (blood-related) adverse events (0.2%) even rarer. In comparison, MuCHIM anticipates just 2 – 6 weeks of therapy for most participants, which would reduce the risk even further. Additionally, symptomatic liver toxicity is very uncommon in people prescribed clarithromycin (3.8 per 100,000 prescriptions). Although clinically significant hepatitis due to these antibiotics is rare, it is further prevented by ensuring that the participant does not consume alcohol or take another liver toxic medication during treatment, and excluding prior viral hepatitis prior to study inclusion. All participants will be instructed to stop antibiotic treatment and to notify the trial team if they develop any symptom of antibiotic-related liver toxicity (abdominal pain, persistent vomiting, or jaundice). Complete recovery of rifampicin- and clarithromycin related hepatitis is expected after stopping treatment.

Toxicity to the ear (ototoxicity) in the form of hearing loss may be a very rare complication of clarithromycin use. Studies in Guinea pigs show that this is reversible. Irreversible hearing loss attributed to clarithromycin appears to be extremely rare, and a number of large studies have not shown any association between clarithromycin (or similar antibiotics) and hearing loss. Nevertheless, this study still evaluates participants for any prior hearing impairment and monitors them closely. Tinnitus (ringing in the ear) is known to be associated with this type of antibiotic, although the few case reports of this complication also suggest it is reversible.

Like a number of medications, clarithromycin may cause an increase in the QTc interval, part of the heart’s electrical activity. The consequences of this are serious but extremely rare. If the QTc interval is significantly prolonged, it may lead to arrhythmia (abnormal heartbeat) which can cause palpitations, dizziness and collapse. Therefore, only people with a low-normal QTc will be eligible to participate in this study. A baseline ECG will exclude pre-existing QTc abnormalities, and will be repeated 1 – 2 weeks into treatment. If the QTc interval has become abnormal, the antibiotics will be stopped.

If either rifampicin or clarithromycin are unable to be continued, either may be replaced by ciprofloxacin (typically dosed at 500 mg orally, twice daily). Like clarithromycin, this antibiotic may also cause an extension in the QTc interval, so if clarithromycin and ciprofloxacin are used in combination, then additional monitoring for QTc prolongation is required with a weekly ECG test.

Ciprofloxacin can cause inflammation in the tendons (tendinopathy) and participants will be instructed to contact study investigators if they develop painful tendons, although risk factors for this (including older age and diabetes) are minimised by careful eligibility criteria. Symptoms such as agitation, restlessness, and confusion have been associated with clarithromycin and ciprofloxacin. Ciprofloxacin will be an alternative only for participants who are unable to tolerate the first combination of antibiotics.

# Expected time to healing and paradoxical reactions

Following antibiotic initiation, most early, limited lesions (≤ 2 cm diameter) heal after an average of 3 months. More rapidly inflamed and/or swollen lesions have been reported in 3.8% of cases in the Bellarine Peninsula, Victoria (in those aged 15 – 60), these may require treatment with oral corticosteroid medication to reduce inflammation. Paradoxical reactions (worsening of lesions while on treatment due to the successful killing of the BU organisms by the antibiotics) are observed after an average of 39 days in approximately one fifth of patients. These are typically mild, and sometimes require corticosteroids (to blunt the immunological response) and/or longer antibiotic treatment in selected cases. As paradoxical reactions give the impression of wound deterioration despite appropriate therapy, participants will be informed of this possibility prior to commencing treatment. These reactions are likely to be less severe in small lesions treated early in healthy young adults. Surgical intervention remains an option for participants who are unable to complete the full duration of antibiotic treatment or for those who prefer to reduce the time to healing.

# Punch biopsy

For non-ulcerative lesions, a minimally invasive biopsy will be performed prior to punch biopsy. At the same study visit, the 3 mm punch biopsy will be obtained to confirm the presence of the bacteria (and compared to the minimally invasive biopsy). For the punch biopsy, a small volume of local anaesthetic will be injected prior to the biopsy to maintain comfort. Allergic reactions from mild to severe may occur in response to any constituent of the local anaesthetic agent (these are extremely rare). The skin will be disinfected prior to biopsy. The risks of biopsy include pain, swelling, bleeding and infection. In the event that an infection occurs, antibiotics will be prescribed for treatment that do not interact with the antibiotic treatment required for BU. The biopsy site will have a wound closure strip and bandage applied, and will heal into a small scar.

# Risk to participant contacts

There are minimal ‘third party’ risks to others, as human-to-human transmission is not thought to occur. Nevertheless, participants with open wounds will receive dressings to cover the wound to minimise environmental contamination. As the study is taking place in Victoria, Australia, where the disease is already endemic (i.e., present in the community), there is no excess risk of introducing the agent into the environment, and there is no conclusive evidence that humans introduce the bacteria into the environment.

# Risk of unexpected participant pregnancy

*M. ulcerans* bacteria are not transmitted from mother to unborn child. Rifampicin is known to reduce the effectiveness of drugs such as the oral contraceptive pill. During the trial and for 30 days after the last dose of any antibiotic, one acceptable method of contraception will be required for people of childbearing potential (Table 1). Participants who become pregnant after the challenge will be monitored and offered treatment should a lesion develop, with prompt surgical excision the favoured treatment option should a lesion develop. In Australia, guidelines recommend the combination of rifampicin and clarithromycin to treat BU in pregnancy. The use of rifampicin is not associated with an increased risk of congenital malformations or adverse pregnancy outcomes. In the final trimester, there is increased risk of haemorrhagic (bleeding) disorders of the newborn, so vitamin K supplementation to the mother is required in the last 4 – 8 weeks of pregnancy to prevent this. Clarithromycin is considered ‘safe to use’ outside of the first trimester by local Australian guidelines, although some research of clarithromycin during pregnancy suggests is may be associated with poor pregnancy-related outcomes. This underscores the importance of screening all candidate participants of childbearing potential for pregnancy at entry and again prior to antibiotic commencement, in addition to ensuring they are aware of the need for acceptable contraception. In the unlikely event that pregnancy occurs during the study, the trial team will collect pregnancy-related information from all pregnant participants, and the participant/s will be followed up to determine the outcome of the pregnancy.

### Table 1: Acceptable methods of contraception for people of childbearing potential

| Sexual abstinence and abstinence from heterosexual intercourse (for people with female sexual reproductive organs) (periodic abstinence and withdrawal methods are not acceptable forms of contraception) |
| --- |
| Male condom and occlusive cap (diaphragm or cervical/vault cap) with spermicidal foam / gel / film / cream / suppository |
| Levonorgestrel-releasing intra-uterine device (IUD) (e.g., Mirena, Skyla) |
| Copper IUD |
| Previous history of bilateral oophorectomy and/or hysterectomy (removal of ovaries and/or uterus) |
| Previous history of bilateral tubal ligation (‘tubes tied’) |
| Vasectomised partner (if sole partner) |

Summary of risks

**Challenge related:**

- Challenge related pain/discomfort
- Redness, swelling, scaling, pustule, nodule, tissue necrosis and/or ulceration at or near challenge site
- Scarring at or near challenge site
- Risk of reaction (rash or other allergic reaction) to any component of the media or preservative (unlikely)
- Anxiety or stigma related to the development of a lesion
- Paradoxical reaction (improvement followed by worsening of wound/pain/swelling)
- Relapse of disease after successful treatment (unlikely)
- Spread to bone, joint or surrounding structures (very unlikely)

**Biopsy or excisional surgery related:**

- Pain/tenderness/redness/swelling/scarring at biopsy/excision site
- Infection at biopsy or surgical site
- Reaction to local anaesthetic (very unlikey)
- Scar

**Monitoring related**

- Pain, tenderness, redness, bruising, swelling, and/or discomfort due to blood tests
- Anaemia due to blood testing (unlikely)
- Discomfort related to providing faecal microbiome sample

**Antibiotic related:**

- Nausea and/or loss of appetite
- Loose or frequent bowel motion
- Bloating and/or dyspepsia
- Abdominal discomfort
- Altered taste sensation
- Discolouration of body fluids (orange/red)
- Tiredness, fatigue, headache, agitation, irritability
- Need to abstain from alcohol during treatment
- Medication interactions, including oral contraceptive pill
- Need to use acceptable birth control method
- Rash or other allergic reaction (rare)
- *C. difficile* diarrhoea (rare)
- Liver function test abnormality (common but mild and rarely clinically important)
- Symptoms of drug related liver inflammation (nausea, vomiting, jaundice) (very rare)
- Prolonged QTc interval on ECG or symptoms related to this (palpitations, dizziness, collapse) (symptoms are very rare)
- Hearing disturbance (very rare)
- Blood disorders (very rare)
