# Supplementary material for: Perceptions of a Buruli ulcer controlled human infection model: How, who, and why?
Source: PLoS Negl Trop Dis. 2025 Feb 5;19(2):e0012593. doi: 10.1371/journal.pntd.0012593 (PMC11819514; doi:10.1371/journal.pntd.0012593)
Supplement: S2 Material — (DOCX) [file pntd.0012593.s002.docx]

**Discusion guide for focus groups**

**1.** Introduction to facilitators and purpose of focus group explained (exploratory nature emphasised), confirm that consent to record session has been obtained, importance and encouragement of participation emphasised (2 – 5 minutes).

**2. Introduction to MuCHIM using PowerPoint slides (15 minutes)**

**3. Discussion on the risks, benefits, perceptions of MuCHIM in general (20 minutes)**

Q1 (*public perceptions*): Has anyone heard of this type of research? What was your first impression?

Q2: Do you think that this issue warrants this type of research?

Q3: (*public perceptions*): Do you think the Australian public will be surprised to hear about the MuCHIM study? What sort of impressions do you expect?
*Exploratory question:* Is there stigma related to this disease in Australia? How will that impact public perceptions of this model?

Q4: (*benefits*): What do you think will motivate people to volunteer for this study?
*Exploratory question*: Do you foresee any other benefits to participating?

*Exploratory question*: Who do you think will be interested in volunteering for this study?

Q5 (*risks*): What are your greatest concerns regarding this study?

Q6. How can we best communicate potential risks to candidate participants?

Q7: What would be the potential consequence if there is inadequate oversight of the trial?

Q8: What do you think is important information to include in the MuCHIM study PICF?

**4. Discussion of protocol aspects (20 minutes)**

Q1: What do you foresee as the greatest potential barriers to implementing this protocol?

Q2 (*consent*): How can we ensure candidate participants understand their involvement in the study?

Q3: What are your thoughts on the inclusion and exclusion criteria? Did anything surprise you?

Q4: What are your impressions of the proposed challenge site location?

Q5: What are your opinions about the possibility of scarring at the challenge site?

Q6: Do you think there will be any concerns or issues with taking antibiotics during this study?

Q7: Do you think participants choose / prefer a surgical approach or antibiotic-only approach?

*Exploratory question*: Why do you think that may be?

Q8: What are your impressions of the protocol’s planned duration of follow up?

*Exploratory question:* Do you think there will be barriers to follow-up?

Q10: We may need to communicate with these participants regularly, for example, to check if they have any issues with their antibiotics. Do you suggest any particular methods of communicating with participants?

**5. Open forum discussion to address/explore other questions, concerns (20 minutes)**

**6. Summary and assessment of level of agreement (2 – 5 min),** invitation to communicate outside of focus group to address private concerns or suggestions.
